# Multimodal Machine Learning‐Based Marker Enables Early Detection and Prognosis Prediction for Hyperuricemia

**DOI:** 10.1002/advs.202404047

**Published:** 2024-07-08

**Authors:** Lin Zeng, Pengcheng Ma, Zeyang Li, Shengxing Liang, Chengkai Wu, Chang Hong, Yan Li, Hao Cui, Ruining Li, Jiaren Wang, Jingzhe He, Wenyuan Li, Lushan Xiao, Li Liu

**Affiliations:** ^1^ Department of Health Management Nanfang Hospital Southern Medical University Guangzhou 510515 China; ^2^ Guangdong Provincial Key Laboratory of Viral Hepatitis Research Department of Infectious Diseases Nanfang Hospital Southern Medical University Guangzhou 510515 China; ^3^ School of Public Health Southern Medical University Guangzhou 510515 China; ^4^ School of Health Management Southern Medical University Guangzhou 510515 China; ^5^ Nanfang Hospital Southern Medical University Guangzhou 510515 China

**Keywords:** gout, hyperuricemia, machine learning, multimodal, prognosis, stratification

## Abstract

Hyperuricemia (HUA) has emerged as the second most prevalent metabolic disorder characterized by prolonged and asymptomatic period, triggering gout and metabolism‐related outcomes. Early detection and prognosis prediction for HUA and gout are crucial for pre‐emptive interventions. Integrating genetic and clinical data from 421287 UK Biobank and 8900 Nanfang Hospital participants, a stacked multimodal machine learning model is developed and validated to synthesize its probabilities as an in‐silico quantitative marker for hyperuricemia (ISHUA). The model demonstrates satisfactory performance in detecting HUA, exhibiting area under the curves (AUCs) of 0.859, 0.836, and 0.779 within the train, internal, and external test sets, respectively. ISHUA is significantly associated with gout and metabolism‐related outcomes, effectively classifying individuals into low‐ and high‐risk groups for gout in the train (AUC, 0.815) and internal test (AUC, 0.814) sets. The high‐risk group shows increased susceptibility to metabolism‐related outcomes, and participants with intermediate or favorable lifestyle profiles have hazard ratios of 0.75 and 0.53 for gout compared with those with unfavorable lifestyles. Similar trends are observed for other metabolism‐related outcomes. The multimodal machine learning‐based ISHUA marker enables personalized risk stratification for gout and metabolism‐related outcomes, and it is unveiled that lifestyle changes can ameliorate these outcomes within high‐risk group, providing guidance for preventive interventions.

## Graphical abstract

This study constructed a novel multimodal machine‐learning model to synthesize an insilico quantitative marker for hyperuricemia (ISHUA). ISHUA enables personalized risk stratification for metabolism‐related outcomes, facilitating stratifying individuals into low‐ and high‐risk groups for incident gout. Further, it is unveiled that metabolism‐related outcomes within high‐risk group can be ameliorated through lifestyle changes, providing valuable insights for effective preventive interventions.

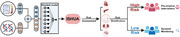

## 1 Introduction

Hyperuricemia (HUA) denotes elevated serum uric acid (SUA) levels attributed to either increased uric acid production or decreased excretion within the body.^[^ ^1^, ^2^ ^]^ Persistent HUA fosters the prolonged deposition of urate crystals, posing potential deleterious effects on joint integrity and exerting a notable impact on individuals' quality of life.^[^ ^3^ ^]^ According to the National Health and Nutrition Examination Survey from 2007 to 2016, HUA prevalence exceeds 20% among both sexes.^[^ ^4^ ^]^ In the Chinese population, a study reported HUA rates of 20.7% in men and 5.6% in women, with prevalence rates steadily increasing annually.^[^ ^5^ ^]^ Ranked as the second most prevalent metabolic disorder after diabetes mellitus,^[^ ^6^ ^]^ HUA has garnered increasing attention as a substantial global public health concern.

Elevated SUA levels not only contribute to gout but also predispose individuals to various metabolic disorders, including chronic kidney disease, hypertension, cardiovascular diseases, and diabetes. Both HUA and gout independently predict premature and all‐cause mortality.^[^ ^7^, ^8^, ^9^, ^10^, ^11^, ^12^, ^13^ ^]^ However, the onset and progression of HUA often manifest subtly, rendering several patients unaware of its latent risks in the absence of symptoms. Although most individuals with HUA remain asymptomatic and do not progress to gout, sophisticated imaging techniques reveal clinically silent urate deposition in ≈ 30% of asymptomatic HUA cases,^[^ ^14^ ^]^ indicating potential for chronic damage. Hence, prompt identification of HUA and early prediction of gout risk may provide invaluable insights for pre‐emptive interventions and prognostic management.

Extensive research has elucidated the impact of clinical factors like age, blood pressure, lipid concentrations, and body mass index (BMI) on SUA levels.^[^ ^15^, ^16^, ^17^ ^]^ Additionally, genetic variations have been shown to contribute significantly, supported by cross‐ethnic Genome‐Wide Association Studies (GWAS) that have identified 183 genetic loci linked to uric acid levels, accounting for 17% of the variance in heritability.^[^ ^8^ ^]^ Furthermore, a subset of these identified genetic variations, identified through GWAS, is located within genes responsible for encoding urate transporters or their regulatory elements.^[^ ^18^, ^19^ ^]^

Currently, risk assessment for HUA or gout predominantly relies on clinical parameters or polygenic risk scores (PRSs),^[^ ^20^, ^21^, ^22^ ^]^ lacking integration into a comprehensive predictive framework that amalgamates genetic and clinical characteristics. Moreover, these existing models typically function as classifiers to predict HUA status in a binary framework rather than quantitatively evaluating the disease on a continuous scale. Quantitative assessment of HUA has the potential to optimize personalized care strategies.

Machine learning (ML), a pivotal branch of artificial intelligence, excels in interpreting vast amounts of data and accurately evaluating complex patterns.^[^ ^23^, ^24^ ^]^ By simulating human brain data processing capabilities, ML significantly enhances accuracy and efficiency compared to traditional methods.^[^ ^25^ ^]^ ML has successfully developed in vitro diagnostic scores for various diseases, including metabolic syndrome^[^ ^26^ ^]^ and coronary artery disease (CAD).^[^ ^27^ ^]^ Multimodal ML integrates information from multiple modalities, reducing biases inherent in single‐modality data and enhancing generalization capabilities.^[^ ^28^ ^]^ Recent advancements in data‐intensive genetic and clinical investigations have facilitated the development of multimodal ML models incorporating genetic and clinical variables for early detection and prognosis prediction.^[^ ^23^, ^29^ ^]^ However, to the best of our knowledge, such tailored models specifically designed for HUA and gout remain absent.

In this study, we aimed to develop and validate a stacked multimodal ML model, incorporating genetic and clinical data, and synthesize the in‐silico quantitative marker for HUA (ISHUA) to enable prompt identification of HUA and early prediction of gout and metabolism‐related outcomes. Additionally, we explored the potential beneficial effects of lifestyle modifications on adverse outcomes.

## 2 Results

### 2.1 Study Workflow

**Figure 1:**
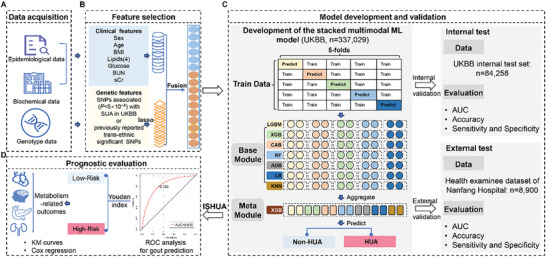
Workflow of the established stacked model and study design. A) Clinical and genetic data acquisition in the UK Biobank and Nanfang Hospital. B) Feature selection performed on the train set and applied to the internal test and external test sets. C) Development and validation of the stacked multimodal machine learning model. D) Prognostic evaluation of the ISHUA in the train and internal test sets. SNP, single nucleotide polymorphism; LASSO, least absolute shrinkage and selection operator; LGBM, Light Gradient Boosting Machine; XGB, classical extreme gradient boosting; CAB, Categorical Boosting; RF, Random Forest; ADB, Adaptive Boosting; LR, Logistic regression; KNN, K‐Nearest Neighbor. AUC, the area under the receiver operating characteristic curve; ISHUA, probabilities as in‐silico scores for hyperuricemia.

The overall study design is illustrated in **Figure** **1**. This study comprised two main components. The first part aimed to train a stacked multimodal ML model using genetic and clinical features extracted from the train set (UK biobank [UKBB], 337029 participants) to predict HUA. Subsequently, the model was validated in internal (UKBB, 84258 participants) and external (Nanfang Hospital cohort, 8900 participants) test sets. The second part involved the construction of ISHUA through the model's probability scores, intended for the quantitative prediction of future risks associated with gout and metabolism‐related outcomes (Table S1, Supporting Information). The effectiveness of ISHUA in the early prediction of heightened gout risk in individuals was assessed. Subsequently, the population was stratified into high‐ and low‐risk groups using the maximum value of the Youden index, enabling further assessment of metabolism‐related outcomes occurrences between these groups.

Lifestyle factors of UKBB dataset, including alcohol consumption, smoking status, physical activity, and diet, were extracted to investigate whether a favorable lifestyle (Table S2, Supporting Information) could mitigate the risk of adverse outcomes related to HUA in the high‐risk group.

### 2.2 Baseline Characteristics of the two Datasets

The demographics and clinical characteristics of all participants are summarized in **Table** **1**. A total of 421287 participants from the UKBB and 8900 from Nanfang Hospital were included for analysis (Figure S1, Supporting Information). The baseline characteristics of the UKBB and Nanfang Hospital cohorts differed. Participants from the UKBB had a lower prevalence of HUA (12.91% vs 38.08%), mainly because participants in Guangdong, China, have been reported to have a high prevalence of HUA.^[^ ^30^ ^]^ Participants in the UKBB cohort were older than those in the Nanfang Hospital cohort and had higher body mass index (BMI) and serum uric acid (sCr), blood urea nitrogen, triglyceride (TG), cholesterol (CHO), low‐density lipoprotein‐cholesterol (LDL‐C), high‐density lipoprotein‐cholesterol (HDL‐C), and blood glucose (Glu) levels. The characteristics of the study participants in the train and internal test sets of the UKBB are presented in Table S3 (Supporting Information). No significant difference was observed in baseline characteristics between the train and internal test sets.

**Table 1:** Baseline characteristics of the UKBB and Nanfang Hospital cohorts.

Characteristic	UK Biobank	Nanfang Hospital	*P*‐value
Number	421 287	8900	
Age, years	58 (50, 63)	36 (30, 46)	< 0.001
Sex, n (%)			0.647
Female	226 725 (53.82)	4812 (54.07)	
Male	194 562 (46.18)	4088 (45.93)	
BMI, kg m^−2^	26.75 (24.16, 29.90)	22.62 (20.47, 25.07)	< 0.001
SUA, umol L^−1^	303.30 (250.7, 361.10)	360.50 (295.00, 438.00)	< 0.001
sCr, umol L^−1^	70.50 (61.40, 81.00)	68.00 (57.00, 81.00)	< 0.001
Urea, mmol L^−1^	5.27 (4.49, 6.14)	4.60 (3.90, 5.40)	< 0.001
TG, mmol L^−1^	1.48 (1.05, 2.15)	1.09 (0.79, 1.62)	< 0.001
CHO, mmol L^−1^	5.65 (4.91, 6.42)	5.02 (4.41, 5.71)	< 0.001
LDL‐C, mmol L^−1^	3.52 (2.95, 4.12)	3.11 (2.65, 3.63)	< 0.001
HDL‐C, mmol L^−1^	1.40 (1.17, 1.67)	1.37 (1.18, 1.58)	< 0.001
Glu, mmol L^−1^	4.93 (4.60, 5.31)	4.77 (4.50, 5.08)	< 0.001
HUA, n (%)	54 401 (12.91)	3389 (38.08)	< 0.001

For continuous features, the median (interquartile range) is reported. For categorical features, count (%) is reported. Continuous variables were assessed using the Mann–Whitney U test. Categorical variables were evaluated using chi‐square or Fisher's exact tests; *P*‐value is used to assess the statistical significance of clinical variables between the UK Biobank and Nanfang Hospital cohorts. BMI, Body mass index; SUA, Serum uric acid; sCr, Serum creatinine; TG, Triglyceride; CHO, Cholesterol; LDL‐C, Low‐density lipoprotein‐cholesterol; HDL‐C, High‐density lipoprotein‐cholesterol; Glu, blood glucose; HUA, hyperuricemia.

### 2.3 Association of Clinical Features with Hyperuricemia and Gout

Regarding the clinical features used in the model, 10 variables were chosen, encompassing sex, age, BMI, TG, CHO, LDL‐C, HDL‐C, Glu, blood urea nitrogen, and sCr. Existing research underscores that age, sex, lipid concentrations, and BMI are significant factors influencing SUA levels.^[^ ^15^, ^16^, ^17^ ^]^ Creatinine and blood urea nitrogen are indicators of renal function, which in turn impacts uric acid excretion. We further explored the effect of clinical features on HUA using logistic regression analyses (Table S4, Supporting Information). Overall, the clinical features showed significant associations with HUA in the UKBB and Nanfang Hospital cohorts, except for CHO, which was significant only in the Nanfang Hospital cohort. Further, Cox proportional hazard regression models demonstrated that these 10 clinical features were associated with an elevated risk of developing gout (Table S5, Supporting Information).

### 2.4 Annotation of Selected SNPs and Enrichment Analysis

In the train set, 1378 SNPs were selected among the 38277 identified as genome‐wide significantly (5 × 10^−8^) associated with SUA in the GWAS analyses in the UKBB^[^ ^31^ ^]^ or associated with SUA in the trans‐ethnic population, as previously reported.^[^ ^8^ ^]^ The selected SNPs were annotated,^[^ ^32^, ^33^ ^]^ mapping into 460 non‐redundant genes. Among these, notable genes included *SLC2A9* (rs3775946), *ABCG2* (rs141471965), *PKD2* (rs139497546), *SLC22A12* (rs111068643), *SLC17A1* (rs1165199), *ADH1C* (rs141973904), *WDR1* (rs10939702), and *NRXN2* (rs572492285). Most of these genes are related to uric acid metabolism or inflammation, and the effects and *P*‐values of the lead SNPs are presented in Table S6 (Supporting Information).

Gene Ontology (GO) and Kyoto Encyclopedia of Genes and Genomes (KEGG) pathway enrichment analyses were conducted. In the KEGG pathway enrichment analysis, nine pathways, including the cholesterol metabolism pathway and type I diabetes mellitus, were found to be significantly enriched (adjusted *P*‐value< 0.05; Figure S2A, Supporting Information). Similarly, GO enrichment analysis revealed enrichment of biological processes such as urate metabolic process, xenobiotic transport, and aorta development; cellular component such as the apical part of cell and apical plasma membrane; molecular function such as active transmembrane transporter activity and insulin−like growth factor I binding (adjusted *p*‐value < 0.05; Figure S2B, Supporting Information).

### 2.5 Performance of the Stacked ML Model in the Train Set

**Figure 2:**
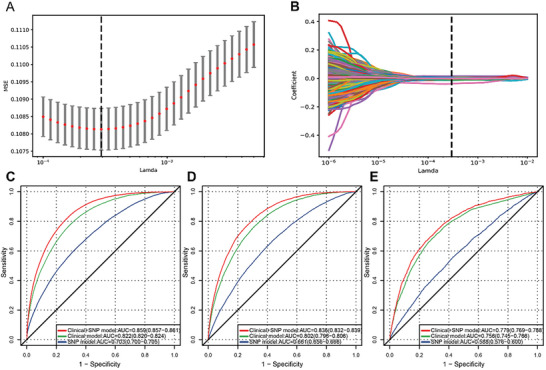
Performance of the stacked ML models for predicting HUA. A) The MSE of different numbers of SNPs revealed by the LASSO model in the train set. A dotted vertical line is drawn at the optimal lambda values by minimum criteria, which is 1378. The lambda min means the lambda at which the minimal MSE is achieved through five‐fold cross‐validation. B) LASSO coefficient profiles of SNPs. C) The ROC analyses for predicting HUA in the train test set with the stacked ML models. D) The ROC analyses for predicting HUA in the internal test set with the stacked ML models. E) The ROC analyses for predicting HUA in the external test set with the stacked ML models. HUA, hyperuricemia; MSE, mean square error; SNP, single nucleotide polymorphism; LASSO, least absolute shrinkage and selection operator; ROC, receiver‐operator characteristic.

By applying the least absolute shrinkage and selection operator (LASSO) algorithm to 38277 genetic variables in the train samples, the most important genetic variables (lambda.min) for identifying HUA were determined. The lambda.min indicates the lambda at which the minimal mean square error was achieved through five‐fold cross‐validation (**Figure** **2**A,B). In total, 1378 genetic features and 10 clinical features were utilized for model construction.

First, we used seven base classifiers in the base module to predict the input features in the train set independently (Figure S3, Supporting Information). Based on the prediction results of the seven base classifiers through five‐fold cross‐validation, we trained the stacked models and observed that the performance was better than that of the individual classifiers. The area under the receiver operating characteristic curve (AUC) and exact values in the train set for base classifiers are presented in Table S7(Supporting Information).

In the train set, the AUC of the stacked model for predicting HUA was 0.703 (95% CI: 0.700, 0.705) using genetic features, 0.822 (95% CI: 0.820, 0.824) using clinical features, and 0.859 (95% CI: 0.857, 0.861) using a combination of genetic and clinical features (Figure 2C). Furthermore, the stacked model, using a combination of genetic and clinical features, predicted HUA with an accuracy of 0.736 (95% CI: 0.735, 0.737), sensitivity of 0.828 (95% CI: 0.825,0.832), and specificity of 0.723 (95% CI: 0.721, 0.724) (Table S8, Supporting Information). Our results showed that the stacked model, which incorporated genetic and clinical features, performed better than the individual classifier.

### 2.6 Performance of Stacked ML Models in the Internal and External Test Sets

We evaluated the stacked ML models using both internal and external test sets. For the internal test set, the AUCs for predicting HUA were 0.661 (95% CI: 0.656, 0.666), 0.802 (95% CI: 0.796, 0.806), and 0.836 (95% CI: 0.832, 0.839) using only genetic features, only clinical features, and combining genetic and clinical features, respectively (Figure 2D). The stacked model, using a combination of genetic and clinical features, predicted HUA with an accuracy of 0.740 (95% CI: 0.737, 0.743), sensitivity of 0.775 (95% CI: 0.768, 0.783), and specificity of 0.734 (95% CI: 0.731, 0.737) (Table S8, Supporting Information).

For the external test set, the AUCs for predicting HUA were 0.588 (95% CI: 0.576, 0.600), 0.756 (95% CI: 0.745, 0.766), and 0.779 (95% CI: 0.769, 0.788) using only genetic features, only clinical features, and combining genetic and clinical features, respectively (Figure 2E). The stacked model, using a combination of genetic and clinical features, predicted HUA with an accuracy of 0.723 (95% CI: 0.714, 0.732), sensitivity of 0.664 (95% CI: 0.648, 0.680), and specificity of 0.759 (95% CI: 0.748, 0.770) (Table S8, Supporting Information). Due to the different age distribution of the Nanfang Hospital cohort compared to the UKBB, we divided the external test set into two age groups (< 40 years and ≥ 40 years) and evaluated the model's performance accordingly (Table S9; Figure S4, Supporting Information). For the stacked model using a combination of genetic and clinical features, the AUC was 0.789 (95% CI: 0.776, 0.801) for participants aged < 40 years and 0.764 (95% CI: 0.748, 0.780) for those aged ≥40 years. Overall, the multimodal model incorporating both genetic and clinical features performed well in both test sets.

### 2.7 Prognostic Evaluation of ISHUA

**Figure 3:**
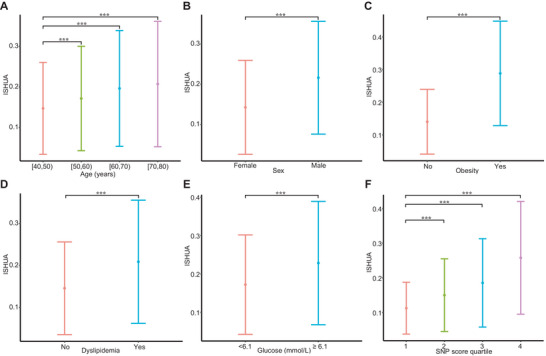
Association of known hyperuricemia risk factors with ISHUA in the train set. ISHUA was evaluated for association with known demographic, clinical and genetic risk factors for hyperuricemia. A) Age, stratified by four decades of age groups. B) Sex, categorized into male and female. C) Obesity, defined as BMI ≥ 30. D) Dyslipidemia, defined based on LDL‐C, CHO, HDL‐C, and TG levels. E) Blood glucose. F) SNP score, derived from the hyperuricemia probabilities of the stacked machine learning model using only genetic features was stratified by quartiles. Data are presented as mean ± standard deviation. Univariate linear regression was used to assess the association between variables and ISHUA: ^**^, *P* < 0.001. ISHUA, insilico score for hyperuricemia; BMI, body mass index; SNP, single nucleotide polymorphisms.

We utilized the HUA probabilities derived from the stacked multimodal model to generate ISHUA for participants in the UKBB train set and assessed its prognostic significance. A correlation was observed between known risk factors for HUA and ISHUA (**Figure** **3**); specifically, ISHUA steadily increased by 0.024 per decade of age (95% CI: 0.024, 0.025; *P* < 0.001), and was higher in males (0.074 [0.073, 0.074]; *P* < 0.001), obese individuals (0.148 [0.147, 0.149]; *P* < 0.001), and those that exhibited dyslipidemia (0.063 [0.062, 0.064]; *P* < 0.001) or dysglycemia (0.056 [0.055, 0.058]; *P* < 0.001) compared to those without these factors. Furthermore, ISHUA captured the risk axes of HUA from the SNP score, increasing by 0.047 per quartile increase in the SNP score (95% CI 0.047–0.048; *P* < 0.001). The results were similar in the internal and external test sets (Figures S5 and S6, Supporting Information).

**Figure 4:**
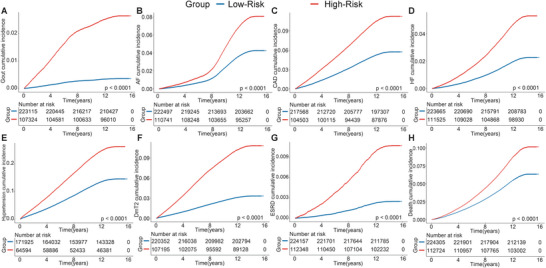
The cumulative risks of developing incident outcomes among the train set, by High‐Risk and Low‐Risk groups. A) Gout, B) AF, C) CAD, D) HF, E) hypertension, F) DmT2, G) ESRD, and H) all‐cause death. The Low‐Risk group was set as the reference group. CAD, coronary artery disease; AF, atrial fibrillation/atrial flutter; HF, heart failure; ESRD, end‐stage renal disease; DmT2, type 2 diabetes mellitus.

After determining the HUA risk captured by ISHUA, we assessed its potential as a quantitative marker for gout and metabolism‐related adverse outcomes. Our results indicated a significant association between ISHUA and the occurrence of metabolic‐related adverse outcomes, particularly gout (Tables S10 and S11, Supporting Information). Therefore, we further evaluated ISHUA's effectiveness in predicting the occurrence of gout. In the train set, ISHUA performed exceptionally in predicting incident gout, with an AUC of 0.815 (95% CI: 0.811, 0.819; Figure S7A, Supporting Information). Based on the largest Youden index, we established an optimal cut‐off value to stratify participants into low‐ (< 0.183) and high‐risk (≥0.183) groups in the train set. Patients in the high‐ and low‐risk groups were estimated to have a heightened risk and low risk for gout occurrence, respectively (**Figure** **4**A).

To examine the generalizability of ISHUA in predicting gout occurrence, we validated and verified the score in the UKBB internal test set. ISHUA maintained good predictive performance in the internal test set, with an AUC of 0.814 (95% CI: 0.806, 0.822) (Figure S7B, Supporting Information). We used the same cut‐off value (0.183) to stratify participants into low‐ and high‐risk groups in the internal test set.

We subsequently evaluated the relationship between the two groups and metabolism‐related adverse outcomes in the train and internal test sets. During a median follow‐up of 13.6 years, we identified 3523, 23353, 10393, 17250, 40730, 18212, 1670, and 24919 incident events of gout, CAD, heart failure (HF), atrial fibrillation/atrial flutter (AF), hypertension, type 2 diabetes mellitus (DmT2), end‐stage renal disease (ESRD), and all‐cause death, respectively, in the train set. We identified 879, 5795, 2588, 4227, 9994, 4570, 416, and 6292 incident events of gout, CAD, HF, AF, hypertension, DmT2, ESRD, and all‐cause death, respectively, in the internal test set (Table S3, Supporting Information). The Kaplan‐Meier survival curve showed that the risk of incident gout or other metabolism‐related outcomes was significantly higher in the high‐risk group in the train and internal test sets (Figure 4; Figure S8, Supporting Information). The high‐risk group was associated with an increased risk of gout, metabolism‐related outcomes, and all‐cause death, after adjusting for lifestyle factors (Tables S12 and S13, Supporting Information).

### 2.8 Association between Lifestyle and Adverse Outcomes in the High‐Risk Group

**Figure 5:**
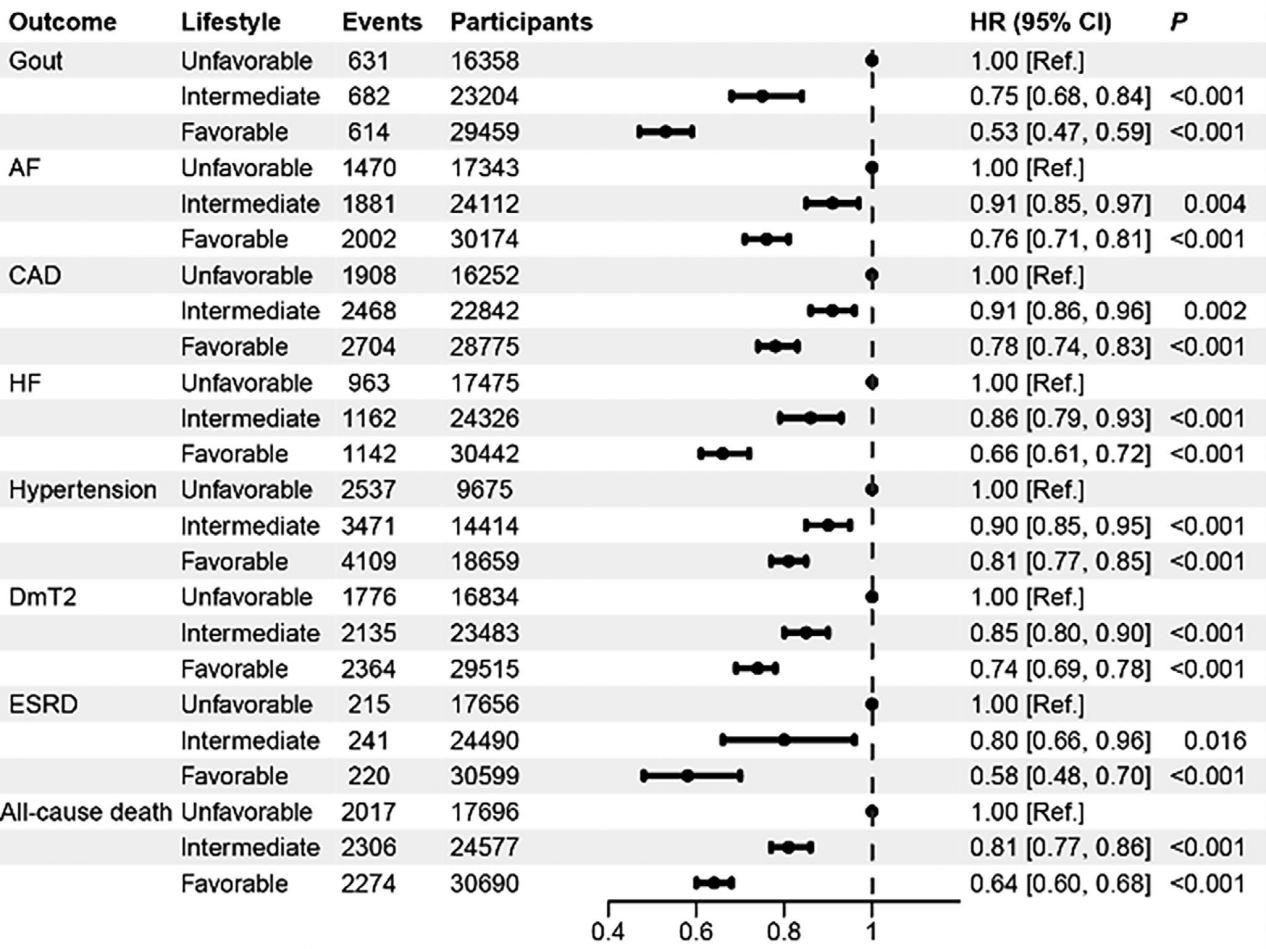
The impact of lifestyle on gout and other outcomes in the High‐Risk group in the train set. The HR values were obtained from Cox proportional hazard regressions. Participants were categorized into three groups according to the number of healthy lifestyle factors: (1) unfavorable lifestyle (0 or 1 healthy lifestyle factors), (2) intermediate lifestyle (2 factors), and (3) favorable lifestyle (3 or 4 factors). HR, hazard ratio; AF, atrial fibrillation/atrial flutter; CAD, coronary artery disease; HF, heart failure; ESRD, end‐stage renal disease; DmT2, type 2 diabetes mellitus.

We subsequently analyzed the association between lifestyle type and outcomes in the high‐risk group to explore whether a favorable lifestyle can mitigate the risk of gout and other metabolism‐related outcomes. In the high‐risk group of the train set, participants with intermediate and favorable lifestyle profiles had lower hazard ratios (HRs) for gout (0.75 [0.68, 0.84] and 0.53 [0.47, 0.59], respectively), AF (0.91 [0.85, 0.97] and 0.76 [0.71, 0.81], respectively), CAD (0.91 [0.86, 0.96] and 0.78 [0.74, 0.83], respectively), HF (0.86 [0.79, 0.93] and 0.66 [0.61, 0.72], respectively), hypertension (0.90 [0.85, 0.95] and 0.81 [0.77, 0.85], respectively), DmT2 (0.85 [0.80, 0.90] and 0.74 [0.69, 0.78], respectively), ESRD (0.80 [0.66, 0.96] and 0.58 [0.48, 0.70], respectively), and all‐cause death (0.81 [0.77, 0.86] and 0.64 [0.60, 0.68], respectively) compared to those with unfavorable lifestyle (**Figure** **5**). Similar trends were observed in the internal test set (Figure S9, Supporting Information).

## 3 Discussion

This study used the large population‐based UKBB cohort as the training and internal test sets, along with a health examinee dataset from Nanfang Hospital as the external test set, to construct a novel stacked multimodal ML model to synthesize an insilico quantitative marker for hyperuricemia (ISHUA). The performance of the proposed model surpassed that of individual monomodal ML models, demonstrating consistent and satisfactory efficacy. The ISHUA marker enables the prediction of metabolic‐related outcomes risk at an early stage, facilitating stratification into low‐ and high‐risk groups for incident gout. Moreover, we revealed that lifestyle changes can mitigate metabolism‐related outcomes in the high‐risk group, providing clinicians with valuable insights for personalized management of HUA and gout.

Several studies have endeavored to develop prognostic models for HUA using clinical features or PRSs respectively. One investigation focused on utilizing clinical data specifically from urban Han Chinese adults to develop a sex‐tailored predictive model for HUA, achieving AUCs of 0.783 (0.779, 0.786) for men and 0.784 (0.778, 0.789) for women.^[^ ^34^ ^]^ Similarly, a separate study applied ML algorithms to forecast SUA status based on routine health examination tests, yielding an optimized AUC of 0.775.^[^ ^20^ ^]^ Additionally, a research effort in Korea conducted GWAS, developing a PRS for SUA, and subsequently formulated a linear regression model for SUA levels by integrating PRS and clinical variables.^[^ ^35^ ^]^ However, this model combined PRS and clinical features without substantiating its efficacy. In contrast to these previous investigations, our study marks the inaugural attempt to use an ML approach and train a stacked multimodal model incorporating genetic and clinical variables, achieving a higher AUC of 0.836 (0.832, 0.839).

In this study, a set of 1378 SNP variants was identified as highly predictive genetic factors contributing to HUA through LASSO analysis. Noteworthy among these variants are rs3775946 and rs1014290, situated within *SLC2A9*, which significantly impact SUA levels. *SLC2A9*, encoding GLUT9, a renal transporter responsible for uric acid reuptake, exhibits profound influence over urate concentrations and gout susceptibility.^[^ ^1^, ^19^ ^]^ Furthermore, the presence of the rs2231142.T allele prompts dysfunction in *ABCG2*, a uric acid transporter primarily located within the gastrointestinal tract and thereby linked to inadequate extra‐renal excretion of uric acid.^[^ ^1^ ^]^ Moreover, the prominence of rs139497546 within *PKD2* emerged as a pivotal factor within the model, demonstrating a positive correlation with *ABCG2* gene expression and suggesting its potential indirect impact on gout development through interactions with *ABCG2*.^[^ ^36^ ^]^ However, the predictive accuracy of the ML model utilizing solely genetic variables was ≈ 0.661. We enhanced the model's predictive efficiency by manually integrating 10 readily available metabolism‐related clinical characteristics, which significantly contribute to and hold biological relevance in HUA.^[^ ^15^, ^30^, ^37^, ^38^, ^39^, ^40^ ^]^ Following the incorporation of metabolism‐related clinical variables into our ML model, the AUC of the internal test set escalated from 0.661 to 0.832, indicating that managing metabolic factors could potentially mitigate HUA risk.

Genetic risk factors contributing to HUA are inherent and persistent from birth. Conversely, clinical risk factors associated with HUA are often responsive to lifestyle choices and amenable to modification. Recent research suggests that specific healthy diets, coupled with weight reduction in overweight or obese individuals, lead to notable improvements in cardiometabolic risk factors and metabolism‐related outcomes.^[^ ^41^ ^]^ Moreover, adopting a healthy lifestyle can partially mitigate the augmented genetic risk of HUA.^[^ ^42^ ^]^ The ISHUA score proposed in this study holds the ability to quantitatively evaluate the metabolism‐related adverse outcomes of HUA on a continuous scale and further stratify participants into low‐ and high‐risk groups based on gout risk. It also demonstrated that adherence to a healthy lifestyle regimen contributed to reducing the incidence of these outcomes among high‐risk individuals.

Integration of the ISHUA score into clinical workflows through electronic health records and clinical decision support systems might enable dynamic monitoring and stratified management of HUA and associated metabolism‐related outcomes during routine health examinations. For high‐risk individuals, enhancing patient education, increasing disease awareness, and promoting lifestyle improvements can potentially prevent occurrences of gout, CAD, and other metabolic adverse outcomes. This approach may alleviate the burden of diseases associated with HUA to a certain extent.

There remains a distance to the practical application of ISHUA, due to several limitations in the study. First, the model was developed in a European cohort and externally tested in a Chinese cohort, potentially introducing a dataset shift. Although performance in the Chinese cohort was acceptable, further validation in diverse populations is necessary. Second, except for gout, the associations of clinical outcomes with ISHUA were only assessed in the UKBB cohort and might not reflect other clinical practices or the general population. Moving forward, we plan to collect follow‐up information from the Nanfang Hospital cohort and evaluate the prognostic value of ISHUA. Third, further prospective studies are crucial to validate the clinical impact of ISHUA, considering factors such as cost‐effectiveness, resource allocation, and acceptance in clinical settings.

## 4 Conclusion

Overall, we established and verified a reliable and practical stacked multimodal ML model trained on genetic and clinical data to synthesize an insilico quantitative marker for HUA, capable of timely identification of HUA and offering potential in personalized risk stratification for gout and metabolism‐related outcomes. Additionally, we demonstrated that lifestyle changes can mitigate adverse consequences in high‐risk individuals. Collectively, the multimodal machine learning‐based ISHUA marker proposed here could offer valuable guidance for dynamic monitoring and precise management of HUA.

## 5 Experimental Section

### Study Population

This study included participants from two cohorts in the UK and China. The UKBB is an ongoing prospective study with clinical and genotype data and multiple follow‐ups from half a million individuals aged 40‐69 years recruited from across the UK between 2006 and 2010. Participants with missing covariate and genotype data were excluded, resulting in a total enrolment of 421287 participants. This cohort was randomly divided in an 8:2 ratio, with 337029 in the train set and 84258 in the internal test set (Figure S1, Supporting Information).

The health examinee dataset of Nanfang Hospital includes information on individuals undergoing health checkups. Data was extracted from individuals aged ≥18 years who visited the hospital between 2015 and 2020. Data collection and preprocessing followed the same criteria as those used in the UKBB. Ultimately, 8900 participants were enrolled in the study, serving as the external test set (Figure S1, Supporting Information).

### Clinical Data and HUA Diagnosis

In the UKBB, clinical data were collected using the corresponding data‐field codes, including demographic information, BMI, SUA, Glu, TG, CHO, LDL‐C, HDL‐C, blood urea nitrogen, and sCr. In the health examinee dataset of Nanfang Hospital, clinical data were retrieved from the electronic health record system. Although no consensus definition of HUA based on the SUA level is available, a HUA diagnosis was made if the SUA level was >420 µmol L^−1^ in men and >360 µmol L^−1^ in women, consistent with previous studies.^[^ ^5^, ^43^ ^]^

### Genotype Data

The genotype data in the UKBB were derived from GWAS ChIP (Affymetrix UK BiLEVE and UK Biobank Axiom arrays). For the health examinee dataset of Nanfang Hospital, genotyping was performed using the Infinium Chinese Genotyping Array v1.0. Genomic DNA was extracted from peripheral blood mononuclear cells. The target genetic variations were SNPs identified as genome‐wide significantly (5×10^−8^) associated with SUA levels in GWAS analyses (http://www.nealelab.is/uk‐biobank) conducted in the UKBB^[^ ^31^ ^]^ or associated with SUA in trans‐ethnic populations as previously reported.^[^ ^8^ ^]^ Genotype information of those SNPs was extracted from the two datasets, and finally, 38277 SNPs simultaneously existing in both datasets were extracted.

### Clinical Outcomes

In the UKBB, detailed follow‐up data of the participants was obtained through their past and future medical and other health‐related records, providing follow‐up information related to cause‐specific mortality and other health events. Cases of gout and metabolism‐related outcomes were identified by the presence of International Classification of Diseases (ICD) codes and self‐reported codes (Table S1, Supporting Information). Metabolism‐related outcomes included hypertension, CAD, HF, AF, ESRD, and DmT2. The follow‐up time for each participant was calculated from baseline until the date when the clinical outcome was identified, lost to follow‐up, or at last follow‐up, whichever occurred first. Cases that occurred before the HUA diagnosis were excluded.

### Assessment of Lifestyle Factors

In the UKBB, information on alcohol consumption, smoking status, and physical activity was obtained from the touchscreen questionnaire, and diet was derived from the Food Frequency Questionnaire. Alcohol consumption was calculated based on self‐reported intake of red wine, white wine, beer, spirits, and fortified wine. Chronic heavy alcohol consumption was defined as ≥3 drinks for women and ≥4 drinks for men on any day (one drink is measured as 8 g ethanol in the UK).^[^ ^44^ ^]^ Smoking status was dichotomized as either smoking or non‐smoking. Physical activity was measured as minutes per week spent walking or engaged in moderate or vigorous activity according to the International Physical Activity Questionnaire. Regular physical activity was defined as engaging in moderate activity for ≥150 min per week, vigorous activity ≥75 min per week, or a combination of moderate and vigorous activity totaling ≥150 min per week.^[^ ^45^ ^]^ Dietary habits were evaluated using the Food Frequency Questionnaire, and a healthy diet score was generated based on intake from seven commonly consumed food groups, aligned with current dietary guidelines for cardiometabolic health.^[^ ^46^ ^]^ A healthy diet was defined as consuming at least four of these seven food groups.^[^ ^46^ ^]^ Table S2 (Supporting Information) provides detailed information on the assessment of healthy lifestyle factors.

In this study, four healthy lifestyle factors: no/moderate alcohol consumption, non‐smoking status, regular physical activity, and adherence to a healthy diet were confirmed. Based on the number of these healthy lifestyle factors, participants were categorized into three groups: 1) unfavorable lifestyle (0 or 1 healthy lifestyle factors), 2) intermediate lifestyle (2 factors), and 3) favorable lifestyle (3 or 4 factors).

### Feature Selection

Feature selection and normalization were performed on the train set from the UKBB and subsequently applied to both internal and external test sets. This process aimed to optimize the performance and clinical interpretability of the ML model while mitigating its complexity. Regarding clinical features, 10 variables with significant impact on SUA levels were chosen based on the existing research, ^[^ ^15^, ^16^, ^17^ ^]^ encompassing sex, age, BMI, TG, CHO, LDL‐C, HDL‐C, glucose, blood urea nitrogen, and sCr. For the genetic features, LASSO regression was utilized to identify the most predictive SNPs associated with the HUA phenotype from the extracted set of 38277 SNPs in the train set (lambda min). LASSO regression analysis was performed using “LassoCV” statistical software (Python Foundation). Given the inherent scale variations between genetic and clinical attributes, all selected features were standardized using “StandardScaler” (Python Foundation). The clinical and genetic features were jointly fed into the stacked model for further analysis (Figure 1A,B).

### Model Development and Validation

For model development, the stacking ML method was employed,^[^ ^47^ ^]^ which was an ensemble learning technique. This approach uses predicted probabilities from individual classifiers (base classifiers) as trainable features for the meta‐classifier. Thus, a stacked multimodal ML architecture was proposed consisting of two components, base and meta modules, which were interconnected in a cascading manner. The base module comprised seven base classifiers, Light Gradient‐Boosting Machine (LGBM), classical extreme Gradient Boosting (XGB), Categorical Boosting (CAB), Random Forest (RF), Adaptive Boosting (ADB), Logistic Regression (LR), and K‐Nearest Neighbor (KNN), all of which operated in parallel, independently predicting input features and subsequently aggregating these predictions. The aggregated results were then transferred to the meta module, which consisted of a meta‐classifier (XGB) that further processed the aggregated results from the base module classifiers to derive the final HUA phenotype prediction (Figure 1C; Figure S3, Supporting Information).

Throughout the training phase, a five‐fold cross‐validation method was utilized in the base module to prepare the inputs for the meta module. This approach helps alleviate overfitting and enhances model stability. This involved randomly dividing the train set into five distinct subsets of the same size for iterative model training (five times in total). Within each iteration, four of the five subsets were concurrently employed for training the seven base classifiers, while the remaining subset was utilized for internal validation purposes. Specifically, the seven base classifiers predicted outcomes on the remaining subset, enabling an evaluation of the base classifiers' performance against ground truth. Simultaneously, these predicted outcomes were aggregated to form input features for the meta‐classifier. Following the completion of the five iterations, an encompassing set of meta input features was derived, enabling the training of the meta classifier using all features to predict HUA phenotype. Subsequently, the stacked model's performance was evaluated on internal and external test sets to assess its efficacy (Figure 1C).

### Prognostic Evaluation

The stacked multimodal model generated probability scores for each participant, which were used as ISHUA values. ISHUA values range from 0 (lowest HUA probability) to 1 (highest HUA probability) and serve as a quantitative marker for HUA, predicting future risks associated with gout and metabolism‐related outcomes.

Using the receiver operating characteristic (ROC) curve analysis, we evaluated the discrimination of ISHUA for gout occurrence in the train set. Then the cut‐off value was applied based on the maximum value of the Youden index to stratify participants into low‐ and high‐risk groups. Subsequently, the association of these risk groups was assessed with metabolism‐related outcomes in participants from the train set.

To validate the discriminatory ability of ISHUA, the probability scores in the internal test set were obtained after testing the stacked multimodal model. The cut‐off value was employed and derived from the train set to categorize the participants of the internal test set into two groups and validate its prognostic value for quantifying the risk of metabolism‐related outcomes (Figure 1D).

### Statistical Analysis

The characteristics of the study participants are presented in Table 1. Continuous variables are presented as medians and interquartile ranges when skewed. Categorical variables are expressed as frequencies and percentages. For comparison between groups, continuous variables were conducted using Mann–Whitney U test. Categorical variables were evaluated using chi‐square or Fisher's exact tests. ROC curve analysis was conducted to assess the prediction efficiency of single and stacked ML models. Kaplan‐Meier curves stratified by ISHUA were generated for gout and metabolism‐related outcomes. Cox proportional hazard regression models were used to examine the association between risk groups (divided by the maximum value of the Youden index of ISHUA for gout) and outcomes. The association between different lifestyle and adverse outcomes in the high‐risk group was assessed using Cox proportional hazard regression models. Effect sizes were reported as HRs and measures of precision (95% confidence intervals [CIs]). All modeling analyses were performed using Python. Other analyses were conducted using R software (version 4.0.2; R Foundation for Statistical Computing, Vienna, Austria). A two‐sided *P*‐value < 0.05 indicated statistical significance for all analyses.

### Ethics Approval

All procedures performed involving human participants were in accordance with the ethical standards of the institutional and/or national research committee and with the 1975 Declaration of Helsinki and its later amendments or comparable ethical standards. Ethical approval was granted for the UK Biobank by the North West‐Haydock Research Ethics Committee (REC reference: 16/NW/0274) and the health examinee dataset of Nanfang Hospital by the Medical Ethics Committee of Nanfang Hospital, Southern Medical University (NFEC‐2019‐161).

## Conflict of Interest

The authors declare no conflict of interest.

## Author Contributions

L.Z., P.M., Z.L., SL., and C.W. contributed equally to this work. L.X. and L.Z. performed conceptualization. L.X. and L.L. performed Resources. L.Z. and P.M. performed the Investigation and methodology. L.Z., P.M., C.W., Z.L., S.L., and C.H. performed data curation. P.M. and L.Z. performed formal analysis and visualization. Z.L., S.L., R.L., and J.H. performed validation. L.Z., Z.L., and Y.L. wrote‐original draft. L.X., J.W., S.L., H.C., and W.L. wrote‐reviewed and edited. L.X. and L.L. performed funding acquisition. L.L. performed supervision. All authors reviewed the manuscript.

## Supporting information

Supporting Information

## Data Availability

The data that support the findings of this study are available from the corresponding author upon reasonable request.

## References

[1] K. Ichida , H. Matsuo , T. Takada , A. Nakayama , K. Murakami , T. Shimizu , Y. Yamanashi , H. Kasuga , H. Nakashima , T. Nakamura , Y. Takada , Y. Kawamura , H. Inoue , C. Okada , Y. Utsumi , Y. Ikebuchi , K. Ito , M. Nakamura , Y. Shinohara , M. Hosoyamada , Y. Sakurai , N. Shinomiya , T. Hosoya , H. Suzuki , Nat. Commun. 2012, 3, 764. 22473008 10.1038/ncomms1756 PMC3337984

[2] N. Dalbeth , A. L. Gosling , A. Gaffo , A. Abhishek , Lancet 2021, 397, 1843. 33798500 10.1016/S0140-6736(21)00569-9

[3] T. J. Major , N. Dalbeth , E. A. Stahl , T. R. Merriman , Nat. Rev. Rheumatol. 2018, 14, 341. 29740155 10.1038/s41584-018-0004-x

[4] M. Chen‐Xu , C. Yokose , S. K. Rai , M. H. Pillinger , H. K. Choi , Arthritis. Rheumatol. 2019, 71, 991. 30618180 10.1002/art.40807 PMC6536335

[5] B. Han , N. Wang , Y. Chen , Q. Li , C. Zhu , Y. Chen , Y. Lu , BMJ Open 2020, 10, e035614. 10.1136/bmjopen-2019-035614 PMC7247391 32439695

[6] Multi‐Disciplinary Expert Task Force on Hyperuricemia and Its Related Diseases, Zhonghua Nei Ke Za Zhi 2017, 56, 235. 10.3760/cma.j.issn.0578-1426.2017.03.021 28253612

[7] T. Bardin , P. Richette , BMC Med. 2017, 15, 123. 28669352 10.1186/s12916-017-0890-9 PMC5494879

[8] A. Tin , J. Marten , V. L. Halperin Kuhns , Y. Li , M. Wuttke , H. Kirsten , K. B. Sieber , C. Qiu , M. Gorski , Z. Yu , A. Giri , G. Sveinbjornsson , M. Li , A. Y. Chu , A. Hoppmann , L. J. O'Connor , B. Prins , T. Nutile , D. Noce , M. Akiyama , M. Cocca , S. Ghasemi , P. J. van der Most , K. Horn , Y. Xu , C. Fuchsberger , S. Sedaghat , S. Afaq , N. Amin , J. Arnlov , et al., Nat. Genet. 2019, 51, 1459. 31578528 10.1038/s41588-019-0504-x PMC6858555

[9] W. T. Crawley , C. G. Jungels , K. R. Stenmark , M. A. Fini , Redox Biol. 2022, 51, 102271. 35228125 10.1016/j.redox.2022.102271 PMC8889273

[10] J. Zhu , Y. Zeng , H. Zhang , Y. Qu , Z. Ying , Y. Sun , Y. Hu , W. Chen , H. Yang , J. Yang , H. Song , Front. Med. (Lausanne) 2021, 8, 817150. 35400029 10.3389/fmed.2021.817150 PMC8985123

[11] H. Yanai , H. Adachi , M. Hakoshima , H. Katsuyama , Int. J. Mol. Sci. 2021, 22, 4416.33922546

[12] Y. Waheed , F. Yang , D. Sun , Korean J. Intern. Med. 2021, 36, 1281. 33045808 10.3904/kjim.2020.340 PMC8588983

[13] R. J. Johnson , G. L. Bakris , C. Borghi , M. B. Chonchol , D. Feldman , M. A. Lanaspa , T. R. Merriman , O. W. Moe , D. B. Mount , L. G. Sanchez Lozada , E. Stahl , D. E. Weiner , G. M. Chertow , Am J. Kidney Dis. 2018, 71, 851. 29496260 10.1053/j.ajkd.2017.12.009 PMC7286363

[14] J. G. Puig , L. M. Beltrán , C. Mejía‐Chew , D. Tevar , R. J. Torres , Nucleosides Nucleotides Nucleic Acids 2016, 35, 517. 27906639 10.1080/15257770.2015.1124999

[15] F. Liu , G. L. Du , N. Song , Y. T. Ma , X. M. Li , X. M. Gao , Y. N. Yang , Lipids Health Dis. 2020, 19, 58. 32238146 10.1186/s12944-020-01211-z PMC7115071

[16] X. B. Huang , W. Q. Zhang , W. W. Tang , Y. Liu , Y. Ning , C. Huang , J. X. Liu , Y. J. Yi , R. H. Xu , T. D. Wang , Sci. Rep. 2020, 10, 15683. 32973308 10.1038/s41598-020-72780-3 PMC7515884

[17] R. Li , L. Zeng , C. Wu , P. Ma , H. Cui , L. Chen , Q. Li , C. Hong , L. Liu , L. Xiao , W. Li , Int. J. Gen. Med. 2022, 15, 2747. 35300131 10.2147/IJGM.S355253 PMC8922363

[18] M. Nakatochi , M. Kanai , A. Nakayama , A. Hishida , Y. Kawamura , S. Ichihara , M. Akiyama , H. Ikezaki , N. Furusyo , S. Shimizu , K. Yamamoto , M. Hirata , R. Okada , S. Kawai , M. Kawaguchi , Y. Nishida , C. Shimanoe , R. Ibusuki , T. Takezaki , M. Nakajima , M. Takao , E. Ozaki , D. Matsui , T. Nishiyama , S. Suzuki , N. Takashima , Y. Kita , K. Endoh , K. Kuriki , H. Uemura , et al., Commun. Biol. 2019, 2, 115. 30993211 10.1038/s42003-019-0339-0 PMC6453927

[19] J. Boocock , M. Leask , Y. Okada , H. Matsuo , Y. Kawamura , Y. Shi , C. Li , D. B. Mount , A. K. Mandal , W. Wang , M. Cadzow , A. L. Gosling , T. J. Major , J. A. Horsfield , H. K. Choi , T. Fadason , J. O'Sullivan , E. A. Stahl , T. R. Merriman , Hum. Mol. Genet. 2020, 29, 923. 31985003 10.1093/hmg/ddaa013

[20] S. Lee , E. K. Choe , B. Park , J. Clin. Med. 2019, 8, 667. 30717373 10.3390/jcm8020172 PMC6406925

[21] D. Ichikawa , T. Saito , W. Ujita , H. Oyama , J. Biomed. Inform. 2016, 64, 20. 27658886 10.1016/j.jbi.2016.09.012

[22] S. Chen , W. Han , L. Kong , Q. Li , C. Yu , J. Zhang , H. He , Food Funct. 2023, 14, 6073. 37318026 10.1039/d3fo01363d

[23] J. G. Greener , S. M. Kandathil , L. Moffat , D. T. Jones , Nat. Rev. Mol. Cell Biol. 2022, 23, 40. 34518686 10.1038/s41580-021-00407-0

[24] X. Chen , W. Shu , L. Zhao , J. Wan , View 2022, 4, 20220038.

[25] D. M. Camacho , K. M. Collins , R. K. Powers , J. C. Costello , J. J. Collins , Cell 2018, 173, 1581. 29887378 10.1016/j.cell.2018.05.015

[26] Y. Chen , W. Xu , W. Zhang , R. Tong , A. Yuan , Z. Li , H. Jiang , L. Hu , L. Huang , Y. Xu , Z. Zhang , M. Sun , X. Yan , A. F. Chen , K. Qian , J. Pu , Cell Rep. Med. 2023, 4, 101109. 37467725 10.1016/j.xcrm.2023.101109 PMC10394172

[27] I. S. Forrest , B. O. Petrazzini , A. Duffy , J. K. Park , C. Marquez‐Luna , D. M. Jordan , G. Rocheleau , J. H. Cho , R. S. Rosenson , J. Narula , G. N. Nadkarni , R. Do , Lancet 2023, 401, 215. 36563696 10.1016/S0140-6736(22)02079-7 PMC10069625

[28] T. Baltrusaitis , C. Ahuja , L. P. Morency , IEEE Trans. Pattern Anal. Mach. Intell. 2019, 41, 423. 29994351 10.1109/TPAMI.2018.2798607

[29] M. Sun , W. Sun , X. Zhao , Z. Li , N. Dalbeth , A. Ji , Y. He , H. Qu , G. Zheng , L. Ma , J. Wang , Y. Shi , X. Fang , H. Chen , T. R. Merriman , C. Li , Arthritis Res. Ther. 2022, 24, 67. 35264217 10.1186/s13075-022-02755-4 PMC8905745

[30] W. Liu , W. Liu , S. Wang , H. Tong , J. Yuan , Z. Zou , J. Liu , D. Yang , Z. Xie , Risk Manag. Healthc. Policy 2021, 14, 655. 33623455 10.2147/RMHP.S293913 PMC7896760

[31] C. Bycroft , C. Freeman , D. Petkova , G. Band , L. T. Elliott , K. Sharp , A. Motyer , D. Vukcevic , O. Delaneau , J. O'Connell , A. Cortes , S. Welsh , A. Young , M. Effingham , G. McVean , S. Leslie , N. Allen , P. Donnelly , J. Marchini , Nature 2018, 562, 203. 30305743 10.1038/s41586-018-0579-z PMC6786975

[32] Y. Lu , C. Quan , H. Chen , X. Bo , C. Zhang , Nucleic Acids Res. 2017, 45, D643. 27789693 10.1093/nar/gkw1022 PMC5210526

[33] S. Purcell , B. Neale , K. Todd‐Brown , L. Thomas , M. A. Ferreira , D. Bender , J. Maller , P. Sklar , P. I. de Bakker , M. J. Daly , P. C. Sham , Am J. Hum. Genet. 2007, 81, 559. 17701901 10.1086/519795 PMC1950838

[34] J. Cao , C. Wang , G. Zhang , X. Ji , Y. Liu , X. Sun , Z. Yuan , Z. Jiang , F. Xue , Int. J. Environ. Res. Public Health 2017, 14, 67. 28085072 10.3390/ijerph14010067 PMC5295318

[35] S. K. Cho , B. Kim , W. Myung , Y. Chang , S. Ryu , H.‐N. Kim , H.‐L. Kim , P.‐H. Kuo , C. A. Winkler , H.‐H. Won , Sci. Rep. 2020, 10, 9179. 32514006 10.1038/s41598-020-66064-z PMC7280503

[36] Z. Dong , J. Zhou , S. Jiang , Y. Li , D. Zhao , C. Yang , Y. Ma , H. He , H. Ji , L. Jin , H. Zou , J. Wang , Hereditas 2020, 157, 2. 32000861 10.1186/s41065-020-0116-6 PMC6986014

[37] Y. L. Hou , X. L. Yang , C. X. Wang , L. X. Zhi , M. J. Yang , C. G. You , Lipids Health Dis. 2019, 18, 81. 30935401 10.1186/s12944-019-1031-6 PMC6444567

[38] L. Zhang , Q. Wan , Y. Zhou , J. Xu , C. Yan , Y. Ma , M. Xu , R. He , Y. Li , X. Zhong , G. Cheng , Y. Lu , Lipids Health Dis. 2019, 18, 147. 31272481 10.1186/s12944-019-1077-5 PMC6611049

[39] N. McCormick , M. J. O'Connor , C. Yokose , T. R. Merriman , D. B. Mount , A. Leong , H. K. Choi , Arthritis Rheumatol. 2021, 73, 2096. 33982892 10.1002/art.41779 PMC8568618

[40] M. Kuwabara , R. Kuwabara , K. Niwa , I. Hisatome , G. Smits , C. A. Roncal‐Jimenez , P. S. MacLean , J. M. Yracheta , M. Ohno , M. A. Lanaspa , R. J. Johnson , D. I. Jalal , Nutrients 2018, 10, 1011. 30081468 10.3390/nu10081011 PMC6115805

[41] C. Yokose , N. McCormick , H. K. Choi , Curr. Opin. Rheumatol. 2021, 33, 135. 33399399 10.1097/BOR.0000000000000779 PMC7886025

[42] T. Zhang , Y. Gu , G. Meng , Q. Zhang , L. Liu , H. Wu , S. Zhang , X. Wang , J. Zhang , S. Sun , X. Wang , M. Zhou , Q. Jia , K. Song , K. Niu , Am J. Med. 2023, 136, 476. 36708795 10.1016/j.amjmed.2023.01.004

[43] Multidisciplinary Expert Task Force on Hyperuricemia and Related Diseases, Chin. Med. J. (Engl) 2017, 130, 2473. 10.4103/0366-6999.216416 PMC5684625 29052570

[44] R. Daviet , G. Aydogan , K. Jagannathan , N. Spilka , P. D. Koellinger , H. R. Kranzler , G. Nave , R. R. Wetherill , Nat. Commun. 2022, 13, 1175. 35246521 10.1038/s41467-022-28735-5 PMC8897479

[45] D. M. Lloyd‐Jones , Y. Hong , D. Labarthe , D. Mozaffarian , L. J. Appel , L. Van Horn , K. Greenlund , S. Daniels , G. Nichol , G. F. Tomaselli , D. K. Arnett , G. C. Fonarow , P. M. Ho , M. S. Lauer , F. A. Masoudi , R. M. Robertson , V. Roger , L. H. Schwamm , P. Sorlie , C. W. Yancy , W. D. Rosamond , Circulation 2010, 121, 586. 20089546 10.1161/CIRCULATIONAHA.109.192703

[46] D. Mozaffarian , Circulation 2016, 133, 187. 26746178 10.1161/CIRCULATIONAHA.115.018585 PMC4814348

[47] A. I. Naimi , L. B. Balzer , Eur. J. Epidemiol. 2018, 33, 459. 29637384 10.1007/s10654-018-0390-z PMC6089257

