# Supplementary material for: Multimodal Machine Learning‐Based Marker Enables Early Detection and Prognosis Prediction for Hyperuricemia
Source: Adv Sci (Weinh). 2024 Jul 8;11(34):2404047. doi: 10.1002/advs.202404047 (PMC11425915; doi:10.1002/advs.202404047)
Supplement: Supplementary file 1 — Supporting Information [file ADVS-11-2404047-s001.docx]

**Supporting Information**

**Multimodal Machine Learning-Based Marker Enables Early Detection and Prognosis Prediction for Hyperuricemia**

*Lin Zeng ^1,2, †^, Pengcheng Ma ^3,4,5, †^, Zeyang Li ^4,5, †^, Shengxing Liang ^3, 5, †^, Chengkai Wu ^3,4, †^, Chang Hong ^2^, Yan Li ^2^, Hao Cui ^2^, Ruining Li ^2^, Jiaren Wang ^2^, Jingzhe He ^2^, Wenyuan Li ^5^, Lushan Xiao ^2,^ * and Li Liu ^1,2,^ **

^1^ Department of Health Management, Nanfang Hospital, Southern Medical University, Guangzhou, 510515, China;

^2^ Guangdong Provincial Key Laboratory of Viral Hepatitis Research, Department of Infectious Diseases, Nanfang Hospital, Southern Medical University, Guangzhou, 510515, China;

^3^ School of Public Health, Southern Medical University, Guangzhou 510515, China;

^4^ School of Health Management, Southern Medical University, Guangzhou 510515, China;

^5^ Nanfang Hospital, Southern Medical University, Guangzhou 510515, China;

^†^These authors contributed equally to this work.

**TABLE OF CONTENTS**

**Supplementary Figures**

**Figure S1.** Flowchart of participant selection in two cohorts. 3

**Figure S2.** Enrichment analysis of gene set of 1,378 selected SNPs in KEGG and GO. 4

**Figure S3.** Illustration of the stacking ensemble method. 5

**Figure S4.** Performance of the stacked ML models for predicting HUA in the age subgroups of external test set. 6

**Figure S5.** Association of known hyperuricemia risk factors with ISHUA in the internal test set. 7

**Figure S6.** Association of known hyperuricemia risk factors with ISHUA in the external test set. 8

**Figure S7.** Performance of the ISHUA for predicting gout. 9

**Figure S8.** The cumulative risks of developing incident outcomes among the internal test set, by High-Risk and Low-Risk groups. 10

**Figure S9.** The impact of lifestyle on Gout and other outcomes in the High-Risk group in the internal test set. 11

**Supplementary Tables**

**Table S1.** Coding Algorithms for Defining diseases in the UK Biobank 12

**Table S2.** Definition of lifestyle factors in the UK Biobank. 13

**Table S3.** Characteristics of study participants in the train and internal test set of UK Biobank. 14-15

**Table S4.** Logistic regression analyses of clinical variables with hyperuricemia in the UK Biobank and Nanfang Hospital cohorts. 16

**Table S5.** Cox proportional hazard regressions of clinical variables with Gout in the UK Biobank. 16

**Table S6.** The lead SNPs associated with serum uric acid screened out by LASSO. 17-19

**Table S7.** Mean performance metrics of base classifiers in the train set across 5-fold cross-validation. 20

**Table S8.** Performance metrics of stacked machine learning model. 21

**Table S9.** Performance metrics of stacked machine learning model in the age subgroups of external test set. 21

**Table S10.** The Associations of ISHUA with gout and other outcomes in the train set. 22

**Table S11.** The Associations of ISHUA with gout and other outcomes in the internal test set. 22

**Table S12.** The Associations of High-Risk group with Gout and other outcomes in the train set. 23

**Table S13.** The Associations of High-Risk group with Gout and other outcomes in the internal test set. 24

**Supplementary Figures
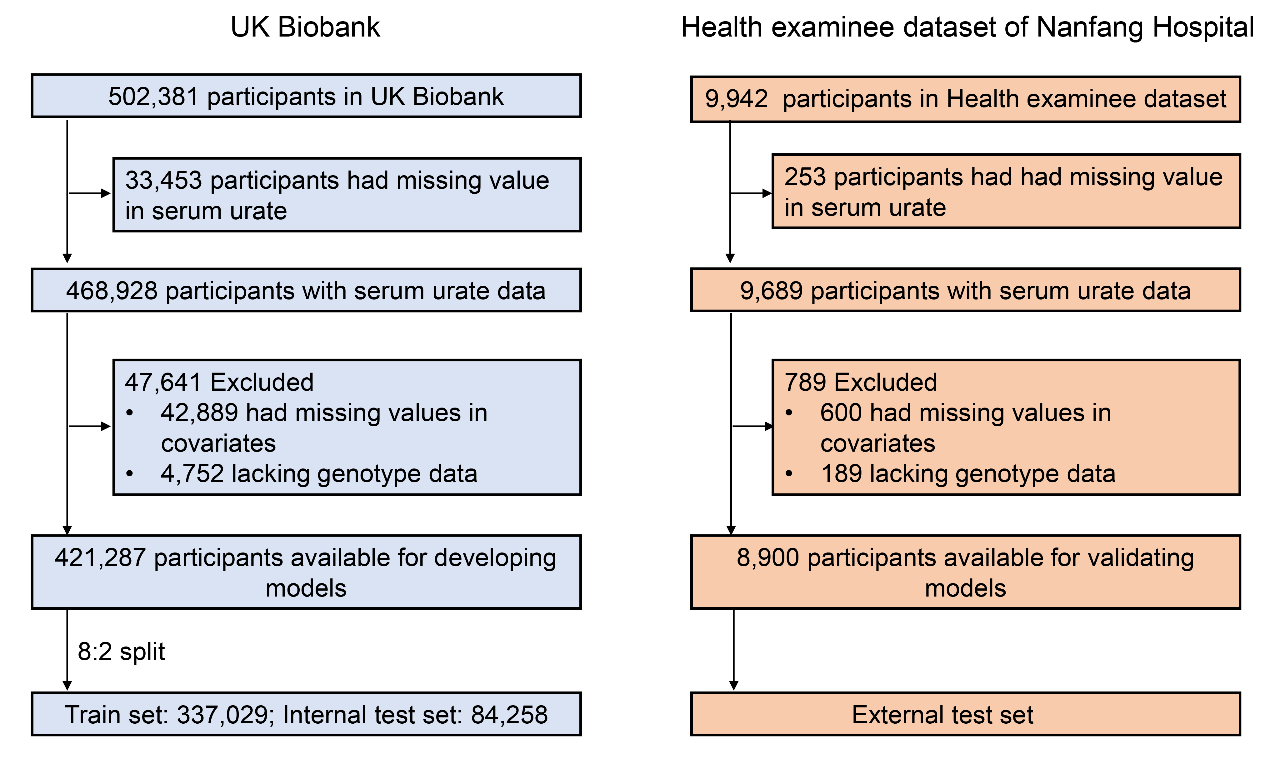
**

**Figure S1.** **Flowchart of participant selection in two cohorts.**


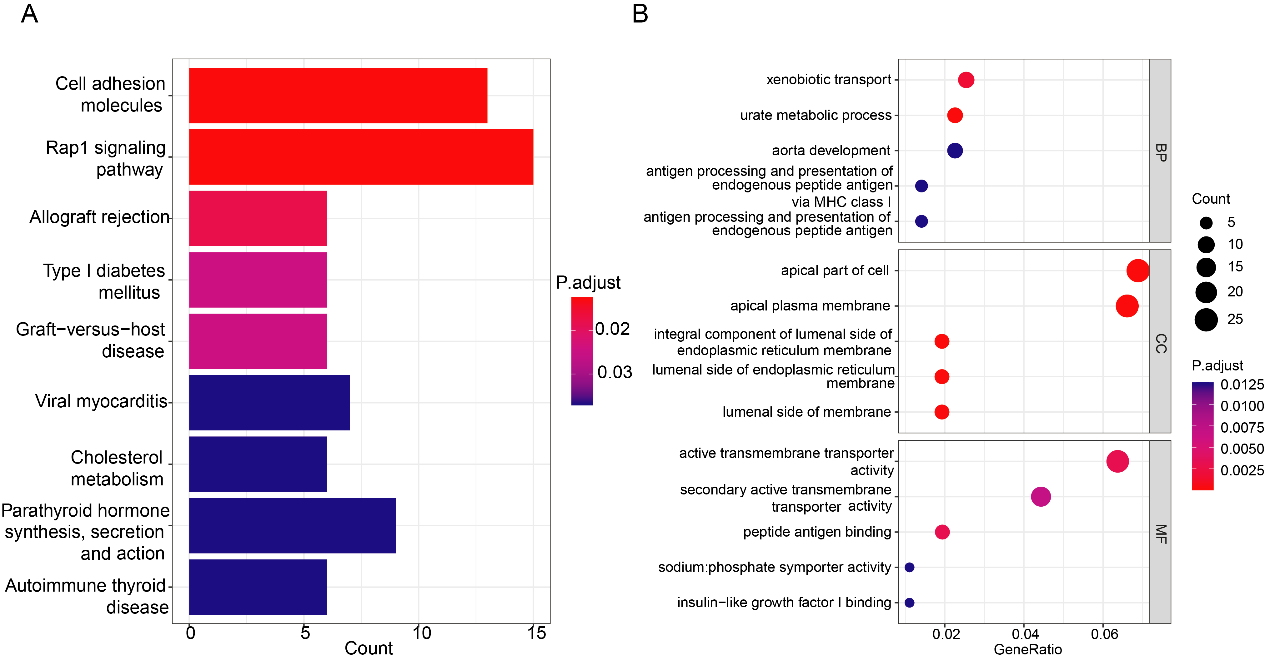


**Figure S2. Enrichment analysis of gene set of 1,378 selected SNPs in KEGG and GO.** A) Enrichment ratio of the nine significant pathways in the KEGG dataset. B) Dot plot of the enrichment analysis of top 6 profiles for BP, CC, and MF respectively in the GO dataset. KEGG, Kyoto Encyclopedia of Genes and Genomes; GO, Gene Ontology; BP, Biological Process; CC, Cellular Component; MF, Molecular Function.

**Figure S3. Illustration of the stacking ensemble method.** LGBM, Light Gradient Boosting Machine; XGB, classical extreme gradient boosting; CAB, Categorical Boosting; RF, Random Forest; ADB, Adaptive Boosting; LR, Logistic regression; KNN, K-Nearest Neighbour.

**
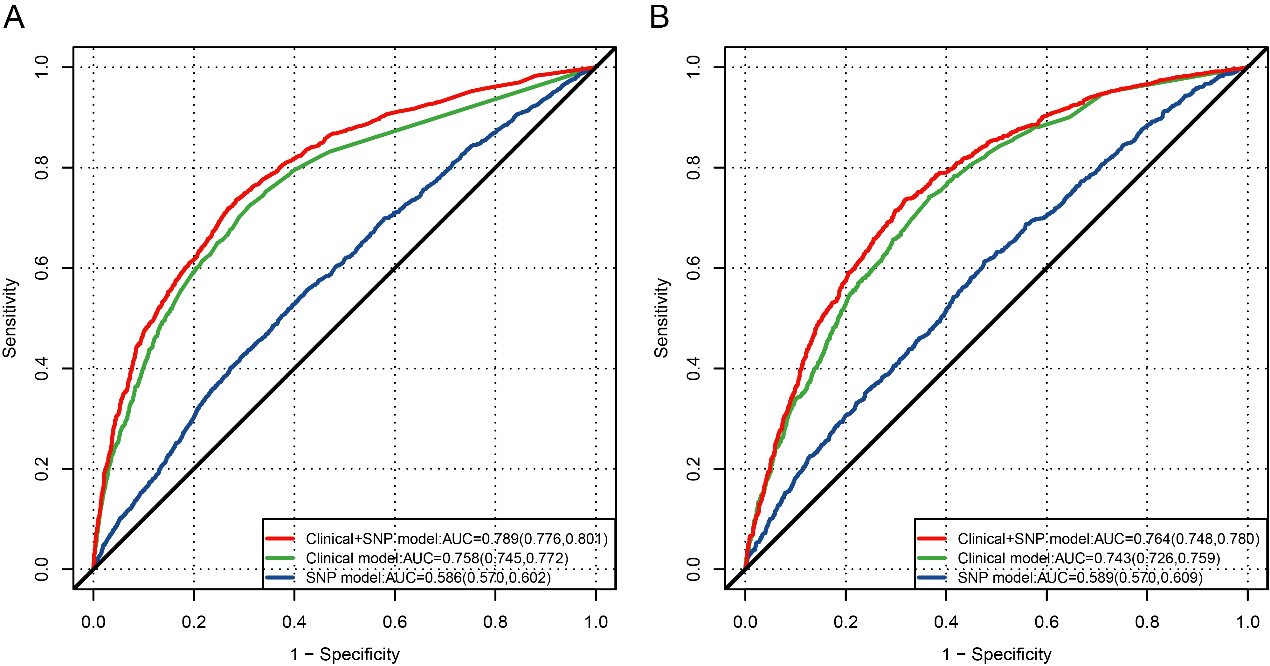
**

**Figure S4.** **Performance of the stacked ML models for HUA in the age subgroups of external test set.** A) The ROC analyses for predicting HUA in the those <40years with the stacked ML models. B) The ROC analyses for predicting HUA in the those ≥ 40years with the stacked ML models; HUA, hyperuricemia; ML, machine learning; ROC, receiver-operator characteristic.

**
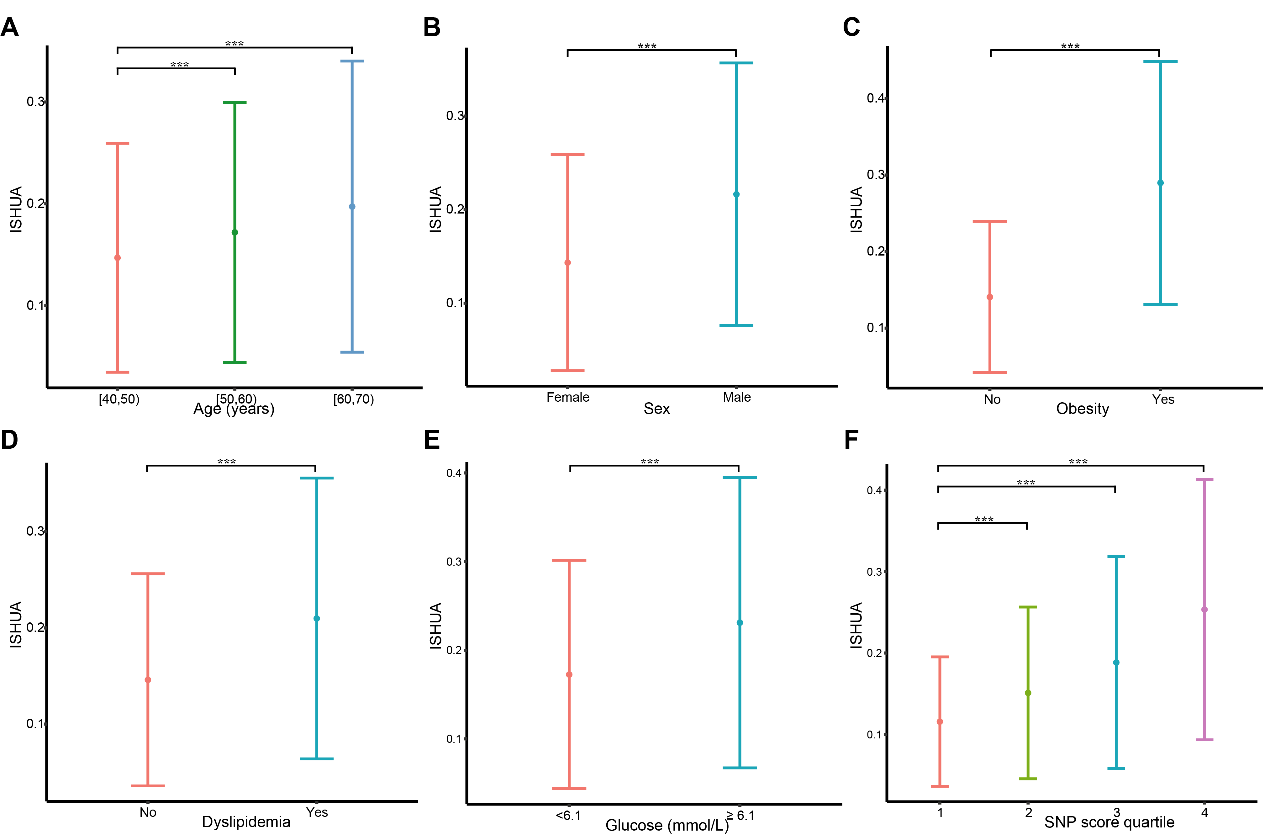
**

**Figure S5. Association of known hyperuricemia risk factors with ISHUA in the internal test set.** ISHUA was evaluated for association with known demographic, clinical and genetic risk factors for hyperuricemia. A) Age, stratified by four decades of age groups. B) Sex, categorized into male and female. C) Obesity, defined as BMI ≥ 30. D) Dyslipidemia, defined based on LDL-C, CHO, HDL-C, TG levels. E) Blood glucose. F) SNP score, derived from the hyperuricemia probabilities of stacked machine learning model using only genetic features and were stratified by quartiles. Data are presented as mean ± standard deviation. Univariate linear regression was used to assess the association between variables and ISHUA: ***, *P* < 0.001. ISHUA, in-silico score for hyperuricemia; BMI, body mass index; SNP, single nucleotide polymorphisms.

ISHUA was associated with known risk factors and genetic risk for hyperuricemia in the internal test set of UK Biobank.

**
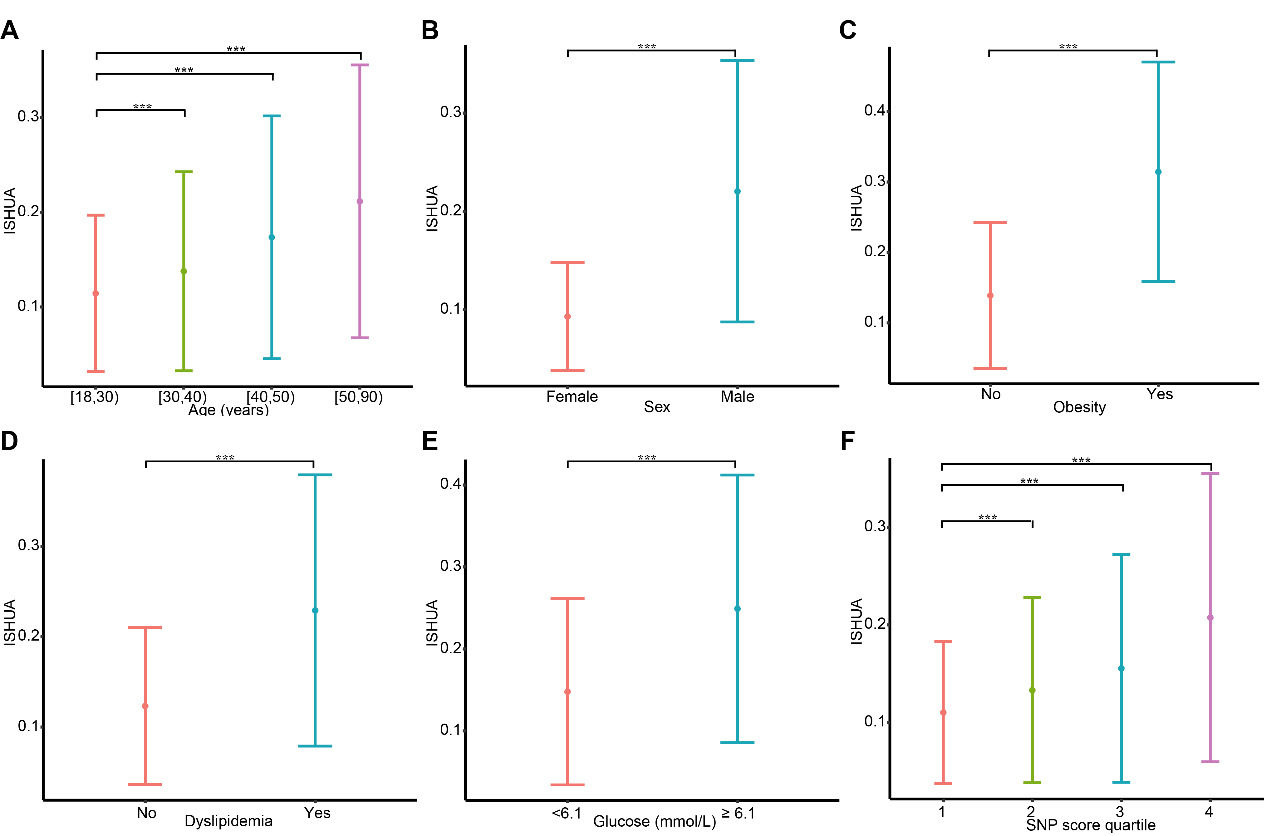
**

**Figure S6. Association of known hyperuricemia risk factors with ISHUA in the external test set.** ISHUA was evaluated for association with known demographic, clinical and genetic risk factors for hyperuricemia. A) Age, stratified by four decades of age groups. B) Sex, categorized into male and female. C) Obesity, defined as BMI ≥ 30. D) Dyslipidemia, defined based on LDL-C, CHO, HDL-C, TG levels. E) Blood glucose. F) SNP score, derived from the hyperuricemia probabilities of stacked machine learning model using only genetic features and were stratified by quartiles. Data are presented as mean ± standard deviation. Univariate linear regression was used to assess the association between variables and ISHUA: ***, *P* < 0.001. ISHUA, in-silico score for hyperuricemia; BMI, body mass index; SNP, single nucleotide polymorphisms.

ISHUA was associated with known risk factors and genetic risk for hyperuricemia in the external test set of Nanfang Hospital cohort.

**
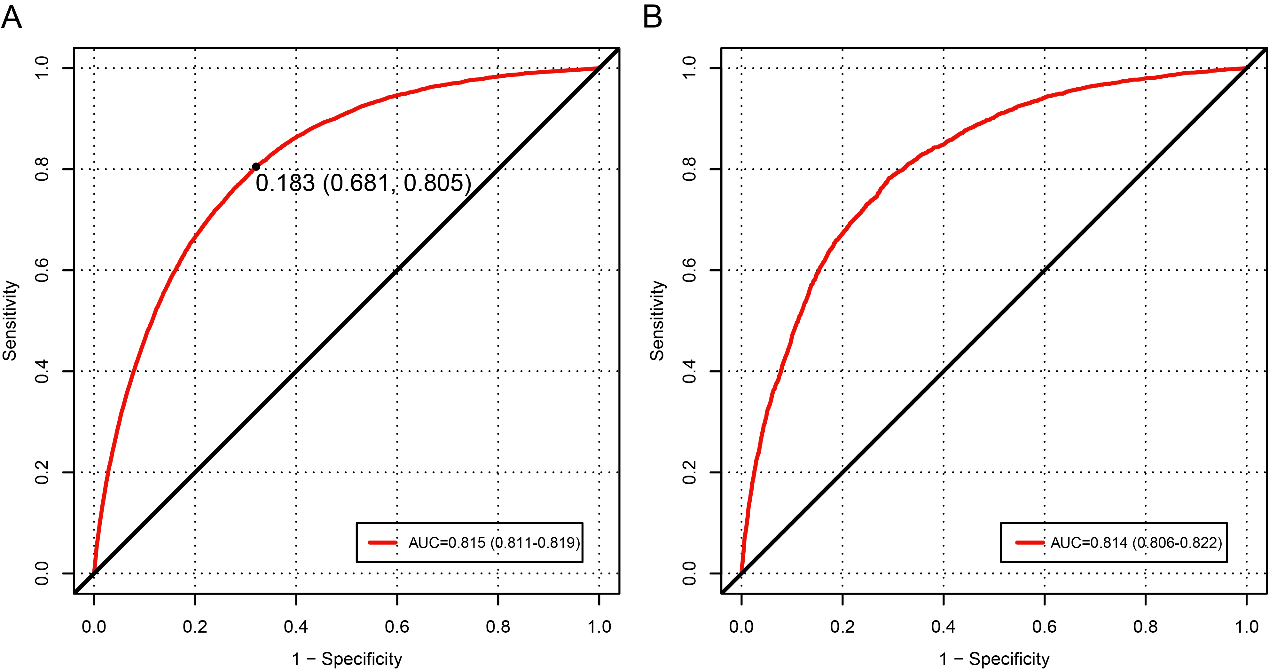
**

**Figure S7. Performance of the ISHUA for predicting gout.** The effectiveness of ISHUA was evaluated for early predicting individuals at heightened gout risk, allowing subsequent stratification of the population into high- and low-risk groups based on the largest Youden index (0.183). A) The ROC analyses for predicting gout in the train test set with ISHUA. B) The ROC analyses for predicting gout in the internal test set with ISHUA. ISHUA, in-silico score for hyperuricemia; ROC, receiver-operator characteristic.

**
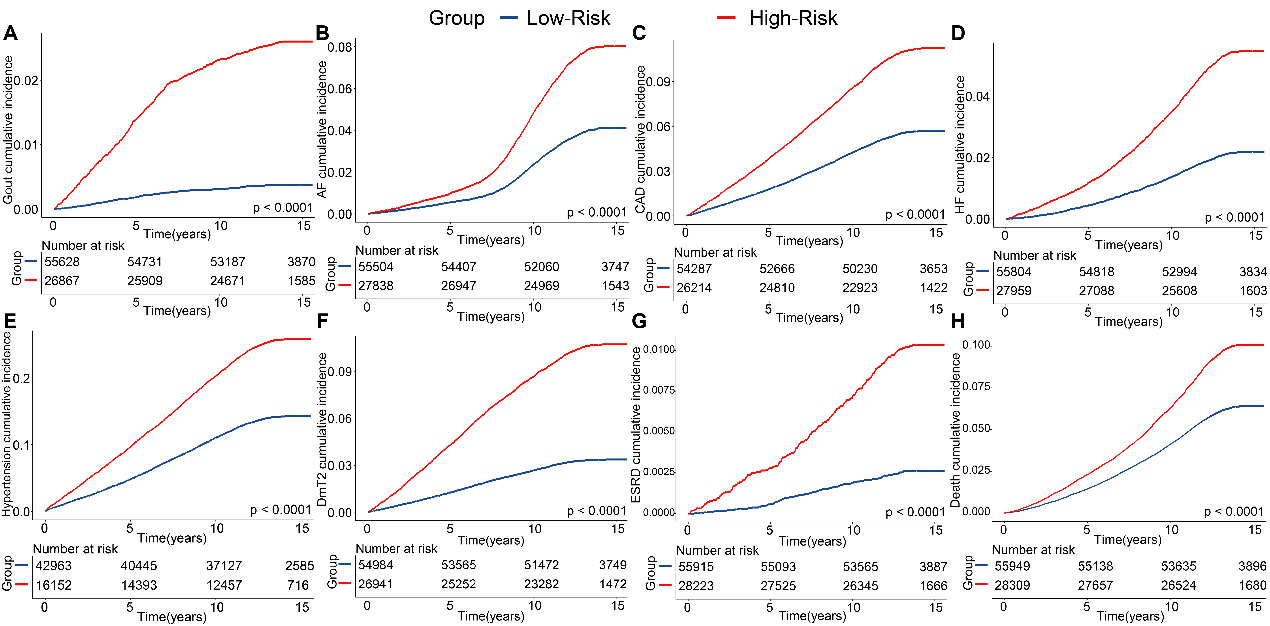
**

**Figure S8. The cumulative risks of developing incident outcomes among the internal test set, by High-Risk and Low-Risk groups.** A) Gout, B) AF, C) CAD, D) HF, E) hypertension, F) DmT2, G) ESRD, and H) all-cause death. Low-Risk group was set as the reference group. CAD, coronary artery disease; AF, atrial fibrillation/atrial flutter; HF, heart failure; ESRD, end-stage renal disease; DmT2, type 2 diabetes mellitus.

**
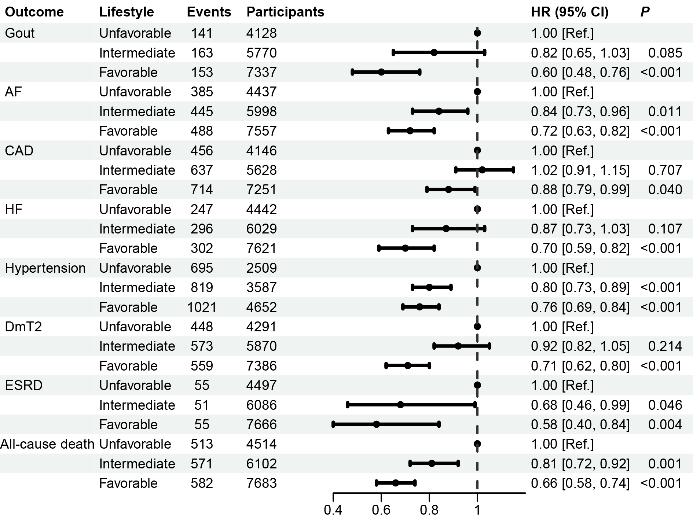
**

**Figure S9. The impact of lifestyle on Gout and other outcomes in the High-Risk group in the internal test set.** The hazard ratio (HR) values were obtained from cox proportional hazard regressions. Participants were categorized into three groups according to the number of healthy lifestyle factors: (1) unfavorable lifestyle (0 or 1 healthy lifestyle factors), (2) intermediate lifestyle (2 factors), and (3) favorable lifestyle (3 or 4 factors). ISHUA, in-silico score for hyperuricemia; AF, atrial fibrillation/atrial flutter; CAD, coronary artery disease; HF, heart failure; ESRD, end-stage renal disease; DmT2, type 2 diabetes mellitus.

**Supplementary Tables**

**Table S1.** **Coding Algorithms for Defining diseases in the UK Biobank.**

| **Diseases** | **ICD-10 code** | **ICD-9 code** | **Self-report*** |
| --- | --- | --- | --- |
| Gout | M10, including sub-codes | 2748, 2749, 274, 2741, 2740 | **Non-cancer illness**: 1466 (Gout) |
| Type 2 Diabetes | E11 | 250, 2500, 2509, 6480, 7902 | **Non-cancer illness**: 1223 (type 2 diabetes)  Exclude: 1222 (Type 1 Diabetes) |
| Hypertension | I10, I11, I12, I13, I15, O10 | 401, 402, 403, 404, 405 | **Medication**: ACE inhibitors, AT-II antagonists, Beta-blockers, Calcium channel-blockers, Thiazide diuretics;  **Non-cancer illness**: 1065 (hypertension), 1072 (essential hypertension) |
| Coronary artery disease | I21, I22, I23, I252, Z951 | 410, 412, 414 | **Non-cancer illness**: 1075 (heart attack/myocardial infarction);  **Operation**: 1070 (coronary angioplasty +/- stent), 1095 (cabg), 1523 (triple heart bypass) |
| Heart failure | I50, I110, I130, I132, Z941, T862 | 428 | **Non-cancer illness**: 1076 (heart failure/pulmonary odema);  **Operation**: 1098 (heart transplant) |
| Atrial fibrillation/Atrial flutter | I48 | 4273 |  |
| End stage renal disease | E853, N165, N180, N185, Q601, T824, T861, Y602, Y612, Y622, Y841, Z490, Z491, Z492, Z940, Z992 | 2273, 5838, 7530, 9961, 9968, E8712, E8722, 585, V560, V420, 9963, 9999, V451, V56, V568, V728 | **Non-cancer illness**: 1193 (renal failure requiring dialysis);  **Operation**:  1195(renal/kidney transplant), 1580(dialysis access surgery), 1581(haemodialysis access / fistula surgery) |

*Self-report data in UK Biobank: Non-cancer illness, data-Field 20002; Treatment/Medication, data-Field 20003; Operation: data-Field 20004.

**Table S2. Definition of lifestyle factors in the UK Biobank.**

| **Factors** | **Unhealthy level** | **Healthy level** |
| --- | --- | --- |
| Alcohol consumption | Heavy alcohol consumption:  women: ≥3 drinks /day  men: ≥4 drinks /day  one drink is measured as 8 g ethanol in the U.K. | Never or moderate consumption:  women: 0 and <3 drinks /day  men: 0 and <4 drinks /day  or never deinked  one drink is measured as 8 g ethanol in the U.K. |
| Smoking status | Smoking | Non-smoking |
| Physical activity | 0-149 min/week of moderate activity OR 0-74 min/week of vigorous activity OR 0-149 min/week of moderate and vigorous activity | ≥150 min/week of moderate activity OR ≥75 min/week of vigorous activity OR ≥150 min/week of moderate and vigorous activity |
| Diet | Intake of 0-3 healthy dietary components:  1. Fruits: ≥ 3 servings/day  2. Vegetables: ≥ 3 servings/day  3. Fish: ≥2 servings/week  4. Processed meats: ≤ 1 serving/week  5.Unprocessed red meats: ≤1.5 servings/week  6. Whole grains: ≥ 3servings/day  7. Refined grains: ≤1.5 servings/day | Intake of ≥ 4 dietary healthy dietary components:  1. Fruits: ≥ 3 servings/day  2. Vegetables: ≥ 3 servings/day  3. Fish: ≥2 servings/week  4. Processed meats: ≤ 1 serving/week  5.Unprocessed red meats: ≤1.5 servings/week  6. Whole grains: ≥ 3servings/day  7. Refined grains: ≤1.5 servings/day |

**Table S3. Characteristics of study participants in the train and internal test set of UK Biobank.**

| **Characteristic** | **Total**  **(N = 421287)** | **Train set**  **(N = 337029)** | **Internal test set**  **(N = 84258)** | ***P*** |
| --- | --- | --- | --- | --- |
| Age, years | 58 (50, 63) | 58 (50, 63) | 58 (50, 63) | 0.544 |
| Sex, n (%) |  |  |  | 0.962 |
| Female | 226725 (53.82) | 181373 (53.82) | 45352 (53.83) |  |
| Male | 194562 (46.18) | 155656 (46.18) | 38906 (46.17) |  |
| BMI, kg/m^2^ | 26.75 (24.16, 29.90) | 26.76 (24.16, 29.90) | 26.74 (24.13, 29.90) | 0.417 |
| SUA, umol/L | 303.3 (250.7, 361.1) | 303.3 (250.8, 361.2) | 303.1 (250.6, 360.8) | 0.772 |
| Hyperuricemia, n (%) | 54401 (12.91) | 43450 (12.89) | 10951 (13.00) | 0.42 |
| sCr, umol/L | 70.5 (61.4, 81.0) | 70.5 (61.4, 80.9) | 70.45 (61.4, 81.0) | 0.565 |
| Urea, mmol/L | 5.27 (4.49, 6.14) | 5.27 (4.49, 6.14) | 5.26 (4.49, 6.14) | 0.527 |
| TG, mmol/L | 1.48 (1.05, 2.15) | 1.48 (1.04, 2.15) | 1.49 (1.05, 2.14) | 0.575 |
| CHO, mmol/L | 5.65 (4.91, 6.42) | 5.65 (4.91, 6.43) | 5.65 (4.91, 6.42) | 0.977 |
| LDL-C, mmol/L | 3.52 (2.95, 4.12) | 3.52 (2.95, 4.12) | 3.52 (2.95, 4.12) | 0.824 |
| HDL-C, mmol/L | 1.40 (1.17, 1.67) | 1.40 (1.17, 1.67) | 1.40 (1.17, 1.67) | 0.819 |
| Glu, mmol/L | 4.93 (4.60, 5.31) | 4.93 (4.60, 5.32) | 4.93 (4.60, 5.31) | 0.587 |
| Smoking status, n (%) | |  |  | 0.314 |
| Smoker | 129179 (30.66) | 103200 (30.62) | 25979 (30.83) |  |
| Non-smoker | 149664 (35.53) | 119906 (35.58) | 29758 (35.32) |  |
| NA | 142444 (33.81) | 113923 (33.80) | 28521 (33.85) |  |
| Alcohol consumption, n (%) | |  |  | 0.224 |
| Excessive | 75790 (17.99) | 60480 (17.95) | 15310 (18.17) |  |
| Never/moderate | 203053 (48.20) | 162626 (48.25) | 40427 (47.98) |  |
| NA | 142444 (33.81) | 113923 (33.80) | 28521 (33.85) |  |
| Physical activity, n (%) | |  |  | 0.866 |
| Inactive | 125638 (29.82) | 100574 (29.84) | 25064 (29.75) |  |
| Active | 153205 (36.37) | 122532 (36.36) | 30673 (36.4) |  |
| NA | 142444 (33.81) | 113923 (33.80) | 28521 (33.85) |  |
| Diet, n (%) ^a^ |  |  |  | 0.702 |
| Unhealthy | 112954 (26.81) | 90293 (26.79) | 22661 (26.89) |  |
| Healthy | 165889 (39.38) | 132813 (39.41) | 33076 (39.26) |  |
| NA | 142444 (33.81) | 113923 (33.80) | 28521 (33.85) |  |
| Lifestyle, n (%) ^b^ |  |  |  | 0.553 |
| Unfavorable | 55088 (13.08) | 43959 (13.04) | 11129 (13.21) |  |
| Intermediate | 87651 (20.81) | 70152 (20.81) | 17499 (20.77) |  |
| Favorable | 136104 (32.31) | 108995 (32.34) | 27109 (32.17) |  |
| NA | 142444 (33.81) | 113923 (33.80) | 28521 (33.85) |  |
| Gout, n (%) |  |  |  | 0.038 |
| No | 408532 (96.97) | 326916 (97) | 81616 (96.86) |  |
| Yes | 4402 (1.04) | 3523 (1.05) | 879 (1.04) |  |
| NA | 8353 (1.98) | 6590 (1.96) | 1763 (2.09) |  |
| CAD, n (%) |  |  |  | 0.848 |
| No | 373424 (88.64) | 298718 (88.63) | 74706 (88.66) |  |
| Yes | 29148 (6.92) | 23353 (6.93) | 5795 (6.88) |  |
| NA | 18715 (4.44) | 14958 (4.44) | 3757 (4.46) |  |
| HF, n (%) |  |  |  | 0.338 |
| No | 405972 (96.36) | 324797 (96.37) | 81175 (96.34) |  |
| Yes | 12981 (3.08) | 10393 (3.08) | 2588 (3.07) |  |
| NA | 2334 (0.55) | 1839 (0.55) | 495 (0.59) |  |
| AF, n (%) |  |  |  | 0.307 |
| No | 395103 (93.78) | 315988 (93.76) | 79115 (93.90) |  |
| Yes | 21477 (5.10) | 17250 (5.12) | 4227 (5.02) |  |
| NA | 4707 (1.12) | 3791 (1.12) | 916 (1.09) |  |
| Hypertension, n (%) |  |  |  | 0.192 |
| No | 244910 (58.13) | 195789 (58.09) | 49121 (58.30) |  |
| Yes | 50724 (12.04) | 40730 (12.09) | 9994 (11.86) |  |
| NA | 125653 (29.83) | 100510 (29.82) | 25143 (29.84) |  |
| DmT2, n (%) |  |  |  | 0.767 |
| No | 386690 (91.79) | 309335 (91.78) | 77355 (91.81) |  |
| Yes | 22782 (5.41) | 18212 (5.40) | 4570 (5.42) |  |
| NA | 11815 (2.80) | 9482 (2.81) | 2333 (2.77) |  |
| ESRD, n (%) |  |  |  | 0.685 |
| No | 418557 (99.35) | 334835 (99.35) | 83722 (99.36) |  |
| Yes | 2086 (0.50) | 1670 (0.50) | 416 (0.49) |  |
| NA | 644 (0.15) | 524 (0.16) | 120 (0.14) |  |
| All-cause death, n (%) | |  |  | 0.469 |
| No | 390076 (92.59) | 312110 (92.61) | 77966 (92.53) |  |
| Yes | 31211 (7.41) | 24919 (7.39) | 6292 (7.47) |  |

^a^ Diet pattern included seven dietary components: fruits, vegetables, whole grains, refined grains, fish, unprocessed meat, and processed meat;

^b^ Lifestyle index was created by four healthy lifestyle factors: never/moderate alcohol consumption, no smoking, regular physical activity, and a healthy diet. Participants were categorized into three groups according to the number of healthy lifestyle factors: (1) unfavorable (0 or 1), (2) intermediate (any 2), and (3) favorable (3 or 4); Abbreviations: HUA, hyperuricemia; BMI, Body mass index; SUA, Serum uric acid; sCr, Serum creatinine; TG, Triglyceride; CHO, Cholesterol; LDL-C, Low-density lipoprotein-cholesterol; HDL-C, High-density lipoprotein-cholesterol; Glu, blood glucose; ISHUA, in-silico score for hyperuricemia; AF, Atrial fibrillation/Atrial flutter; CAD, coronary artery disease; HF, heart failure; DmT2, type 2 diabetes mellitus; ESRD, end-stage renal disease;

**Table S4. Logistic regression analyses of clinical variables with hyperuricemia in the UK Biobank and Nanfang Hospital cohorts.**

|  | **UK Biobank** | | **Nanfang Hospital** | |
| --- | --- | --- | --- | --- |
|  | **OR [95%CI]** | ***P*-value** | **OR [95%CI]** | ***P*-value** |
| Age, years | 1.03 [1.03-1.03] | < 0.001 | 1.02 [1.01-1.02) | < 0.001 |
| Sex (Male vs. Female) | 2.00 [1.97-2.04] | < 0.001 | 4.27 [3.89-4.67] | < 0.001 |
| BMI, kg/m^2^ | 1.15 [1.15-1.15] | < 0.001 | 1.26 [1.24-1.28] | < 0.001 |
| sCr, umol/L | 1.04 [1.04-1.04] | < 0.001 | 1.05 [1.05-1.06] | < 0.001 |
| Urea, mmol/L | 1.43 [1.42-1.44] | < 0.001 | 1.37 [1.32-1.42] | < 0.001 |
| TG, mmol/L | 1.58 [1.56-1.59] | < 0.001 | 1.95 [1.84-2.07] | < 0.001 |
| LDL-C, mmol/L | 1.06 [1.05-1.07] | < 0.001 | 1.70 [1.60-1.80] | < 0.001 |
| HDL-C, mmol/L | 0.26 [0.26-0.27] | < 0.001 | 0.24 [0.20-0.28] | < 0.001 |
| CHO, mmol/L | 1.00 [1.00-1.01] | 0.396 | 1.42 [1.35-1.48] | < 0.001 |
| Dyslipidemia | 1.95 [1.91-1.99] | < 0.001 | 2.68 [2.43-2.95] | < 0.001 |
| Glu, mmol/L | 1.10 [1.09-1.11] | < 0.001 | 1.16 [1.10-1.22] | < 0.001 |

Dyslipidemia is defined based on the presence of one or more of the following criteria: LDL-C ≥ 4.1 mmol/L, CHO ≥ 6.2 mmol/L, HDL-C < 1.0 mmol/L, or TG ≥ 2.3 mmol/L.BMI, Body mass index; sCr, Serum creatinine; TG, Triglyceride; CHO, Cholesterol; LDL-C, Low-density lipoprotein-cholesterol; HDL-C, High-density lipoprotein-cholesterol; Glu, blood glucose.

**Table S5. Cox proportional hazard regressions of clinical variables with Gout in the UK Biobank.**

|  | HR [95%CI] | *P*-value |
| --- | --- | --- |
| Age, years | 1.05 [1.04, 1.05] | <0.001 |
| Sex (Male vs. Female) | 4.37 [4.07, 4.69] | <0.001 |
| BMI, kg/m^2^ | 1.09 [1.09, 1.10] | <0.001 |
| TG | 1.41 [1.38, 1.43] | <0.001 |
| LDL-C | 0.84 [0.81, 0.87] | <0.001 |
| CHO | 0.85 [0.83, 0.87] | <0.001 |
| HDL-C | 0.25 [0.23, 0.27] | <0.001 |
| Dyslipidemia | 1.61 [1.51, 1.71] | <0.001 |
| sCr, umol/L | 1.01 [1.01, 1.01] | <0.001 |
| Urea, mmol/L | 1.27 [1.26, 1.28] | <0.001 |
| Glu, mmol/L | 1.09 [1.07, 1.11] | <0.001 |

Dyslipidemia is defined based on the presence of one or more of the following criteria: LDL-C ≥ 4.1 mmol/L, CHO ≥ 6.2 mmol/L, HDL-C < 1.0 mmol/L, or TG ≥ 2.3 mmol/L.BMI, Body mass index; sCr, Serum creatinine; TG, Triglyceride; CHO, Cholesterol; LDL-C, Low-density lipoprotein-cholesterol; HDL-C, High-density lipoprotein-cholesterol; Glu, blood glucose.

**Table S6.** **The lead SNPs associated with serum uric acid screened out by LASSO.**

| **Rsid** | **Linear closest gene*** | **Minor allele** | **Beta** | **SE** | ***P*-value** |
| --- | --- | --- | --- | --- | --- |
| rs6810736 | SLC2A9 | C | 7.1162 | 0.16986 | <5.94E-259 |
| rs6824636 | SLC2A9 | C | 8.6143 | 0.1858 | <5.94E-259 |
| rs12644592 | SLC2A9 | G | 8.4502 | 0.17683 | <5.94E-259 |
| rs11722228 | SLC2A9 | T | 13.008 | 0.17142 | <5.94E-259 |
| rs10805346 | SLC2A9 | C | -16.18 | 0.16349 | <5.94E-259 |
| rs16890979 | SLC2A9 | T | -23.47 | 0.18746 | <5.94E-259 |
| rs62294282 | SLC2A9 | A | 22.744 | 0.18092 | <5.94E-259 |
| rs6843466 | SLC2A9 | T | 21.651 | 0.18445 | <5.94E-259 |
| rs11723439 | SLC2A9 | T | -24.751 | 0.2018 | <5.94E-259 |
| rs3775948 | SLC2A9 | G | 22.816 | 0.18711 | <5.94E-259 |
| rs3775946 | SLC2A9 | A | 23.289 | 0.19 | <5.94E-259 |
| rs4529048 | SLC2A9 | C | 22.761 | 0.18724 | <5.94E-259 |
| rs1014290 | SLC2A9 | G | 23.035 | 0.18787 | <5.94E-259 |
| rs11557743 | WDR1 | G | -11.514 | 0.16419 | <5.94E-259 |
| rs141471965 | ABCG2 | T | 14.147 | 0.25628 | <5.94E-259 |
| rs2231142 | ABCG2 | T | 14.14 | 0.25625 | <5.94E-259 |
| rs2622609 | ABCG2 | C | 6.2193 | 0.16524 | <5.94E-259 |
| rs28692745 | ABCG2 | A | -5.7036 | 0.16578 | 5.94E-259 |
| rs11732936 | ABCG2 | G | 5.6978 | 0.16578 | 1.91E-258 |
| rs2622627 | ABCG2 | C | 5.6433 | 0.16422 | 2.356E-258 |
| rs3114018 | ABCG2 | A | 5.4809 | 0.16419 | 6.173E-244 |
| rs2622621 | ABCG2 | G | 5.7608 | 0.17342 | 1.422E-241 |
| rs1165199 | SLC17A1 | C | 5.3793 | 0.16531 | 6.232E-232 |
| rs1165195 | SLC17A1 | T | 5.3782 | 0.16532 | 8.362E-232 |
| rs56223908 | SLC2A9 | C | 9.5869 | 0.29602 | 9.736E-230 |
| rs75110987 | SLC17A1 | A | 5.3591 | 0.16631 | 1.838E-227 |
| rs16869060 | CLNK | G | -5.4986 | 0.17499 | 2.028E-216 |
| rs12209125 | SLC17A4 | C | -5.102 | 0.1671 | 1.786E-204 |
| rs3109821 | ABCG2 | A | 5.0703 | 0.17036 | 2.094E-194 |
| rs10939702 | WDR1 | T | 4.7649 | 0.16671 | 1.787E-179 |
| rs139497546 | PKD2 | CATATAT | 8.3588 | 0.29496 | 1.843E-176 |
| rs771556650 | ABCG2 | A | -4.9494 | 0.17592 | 5.755E-174 |
| rs28643326 | SLC2A9 | C | 5.182 | 0.19656 | 5.124E-153 |
| rs7929308 | SLC22A11 | T | -4.3293 | 0.16511 | 2.184E-151 |
| rs17300741 | SLC22A11 | A | -4.3209 | 0.165 | 5.141E-151 |
| rs6456703 | SLC17A1 | T | 4.8963 | 0.18814 | 3.6E-149 |
| rs57827982 | SLC22A11 | T | -4.2223 | 0.16574 | 5.011E-143 |
| rs6913879 | SLC17A1 | C | 4.7182 | 0.18536 | 8.604E-143 |
| rs9461218 | SLC17A1 | A | 4.6918 | 0.1891 | 9.053E-136 |
| rs11729986 | LOC101928948 | G | 4.3949 | 0.17915 | 8.818E-133 |
| rs1260326 | GCKR | T | -3.9953 | 0.16765 | 2.032E-125 |
| rs2328893 | SLC17A4 | A | -4.2361 | 0.17776 | 2.047E-125 |
| rs542984928 | SLC22A12 | CT | 5.5523 | 0.23527 | 4.889E-123 |
| rs1171616 | SLC16A9 | G | 4.481 | 0.19429 | 1.336E-117 |
| rs9684707 | LOC101928948 | G | 4.1362 | 0.18037 | 2.736E-116 |
| rs780093 | GCKR | T | -3.7795 | 0.16857 | 2.998E-111 |
| rs35342884 | SLC2A9 | C | -4.5457 | 0.20538 | 1.799E-108 |
| rs6456708 | HIST1H2AB, HIST1H3B | G | 3.4665 | 0.16481 | 3.7116E-98 |
| rs7929627 | SLC22A12 | G | 4.3095 | 0.2094 | 4.7343E-94 |
| rs7121121 | NRXN2 | C | 4.2919 | 0.20927 | 2.0166E-93 |
| rs530252 | NRXN2 | C | 4.2834 | 0.20923 | 4.2888E-93 |
| rs516117 | NRXN2 | C | 4.2832 | 0.20924 | 4.467E-93 |
| rs572492285 | NRXN2 | CA | 4.6197 | 0.22612 | 1.0227E-92 |
| rs2229357 | INHBC | A | -3.8521 | 0.19073 | 1.171E-90 |
| rs11736552 | ABCG2 | G | 3.484 | 0.17365 | 1.7341E-89 |
| rs540730 | R3HDM2 | T | 3.7672 | 0.18998 | 1.8392E-87 |
| rs551623654 | R3HDM2 | GT | -3.7422 | 0.19088 | 1.574E-85 |
| rs11614506 | R3HDM2 | C | -3.714 | 0.19542 | 1.6887E-80 |
| rs5792371 | OVOL1, OVOL1-AS1, LOC101927828 | TG | 3.1771 | 0.17276 | 1.713E-75 |
| rs2869736 | ABCG2 | T | -2.9448 | 0.1653 | 5.8591E-71 |
| rs423144 | THBS3, LOC101928155 | T | -2.9124 | 0.16554 | 2.9385E-69 |
| rs111915606 | SLC17A3 | T | -4.034 | 0.23313 | 4.717E-67 |
| rs1285875 | RREB1 | G | -3.165 | 0.18785 | 1.1483E-63 |
| rs537596349 | R3HDM2 | A | -4.0426 | 0.24038 | 1.9201E-63 |
| rs555730055 | R3HDM2 | A | -4.0426 | 0.24038 | 1.9201E-63 |
| rs642803 | OVOL1, LOC101927828 | T | -2.7605 | 0.16441 | 3.0837E-63 |
| rs760077 | THBS3, MTX1 | A | -2.7959 | 0.16754 | 1.6927E-62 |
| rs10994860 | A1CF | T | 3.5918 | 0.21523 | 1.6932E-62 |
| rs111068643 | SLC22A12 | T | 7.4235 | 0.44801 | 1.2128E-61 |
| rs4971099 | TRIM46 | G | -2.728 | 0.16504 | 2.3842E-61 |
| rs56265379 | SLC22A12 | C | 7.2979 | 0.44944 | 2.8706E-59 |
| rs12664474 | SLC17A3 | G | 3.6297 | 0.22378 | 3.8248E-59 |
| rs559803064 | SFMBT1 | TA | 2.637 | 0.1662 | 1.1329E-56 |
| rs4697758 | CLNK | A | -3.636 | 0.22928 | 1.2866E-56 |
| rs1334577 | RREB1 | A | 3.0424 | 0.19305 | 6.2108E-56 |
| rs1413700 | RREB1 | C | 3.0331 | 0.19304 | 1.2979E-55 |
| rs17762454 | RREB1 | T | 2.8727 | 0.18652 | 1.6649E-53 |
| rs12908437 | IGF1R | T | -2.6018 | 0.17084 | 2.3711E-52 |
| rs113770973 | SFMBT1 | CA | 2.6327 | 0.17398 | 1.0375E-51 |
| rs10636318 | HIST1H4C | GA | -2.7287 | 0.18084 | 1.976E-51 |
| rs2941484 | HNF4G | T | 2.5029 | 0.16588 | 2.004E-51 |
| rs13082026 | SFMBT1 | T | 2.5709 | 0.17148 | 8.6095E-51 |
| rs4886755 | NRG4 | A | 2.4487 | 0.16399 | 2.1155E-50 |
| rs2581806 | SFMBT1 | G | 2.5962 | 0.17549 | 1.6566E-49 |
| rs496388 | OVOL1 | T | -2.5181 | 0.17075 | 3.2988E-49 |
| rs2941471 | HNF4G | G | 2.4317 | 0.16536 | 6.1218E-49 |
| rs2943540 | HNF4G | T | 2.4297 | 0.16549 | 8.7407E-49 |
| rs2725223 | PKD2 | T | 2.4323 | 0.16614 | 1.6058E-48 |
| rs6933716 | RREB1 | T | -2.5888 | 0.17694 | 1.8485E-48 |
| rs564438186 | LOC101929118 | TA | 2.9678 | 0.20468 | 1.2567E-47 |
| rs34455506 | ABCG2 | A | -2.4723 | 0.171 | 2.308E-47 |
| rs59350108 | IGF1R | C | 2.4998 | 0.1737 | 6.0147E-47 |
| rs112232020 | SFMBT1 | A | -2.4602 | 0.17134 | 9.6983E-47 |
| rs59646751 | IGF1R | T | 2.4775 | 0.17784 | 4.2191E-44 |
| rs35368205 | MLXIPL | T | -2.84 | 0.20452 | 7.901E-44 |
| rs72734547 | UBE2Q2 | T | 2.2803 | 0.1682 | 7.3466E-42 |
| rs17145750 | MLXIPL | T | -3.0148 | 0.2225 | 8.1504E-42 |
| rs35332062 | MLXIPL | A | -3.2816 | 0.24479 | 5.7384E-41 |
| rs11684134 | GTF3C2, GTF3C2-AS1, LOC100505624 | G | -2.2547 | 0.16824 | 6.0761E-41 |
| rs900347 | PDZK1 | A | -2.2287 | 0.16738 | 1.9475E-40 |
| rs17014016 | LOC101929118 | A | -2.1958 | 0.16506 | 2.2683E-40 |
| rs4704054 | TMEM171 | G | -2.3151 | 0.17531 | 8.3114E-40 |
| rs2990245 | GBAP1 | C | -2.1577 | 0.16406 | 1.6858E-39 |
| rs80051818 | EIF2B4, SNX17 | C | -2.2462 | 0.17501 | 1.0666E-37 |
| rs3829578 | HLF | T | -2.1309 | 0.1685 | 1.194E-36 |
| rs2004649 | MAP3K11 | A | 2.0792 | 0.16575 | 4.3593E-36 |
| rs1545207 | LOC101929118 | A | 2.2719 | 0.1834 | 3.0916E-35 |
| rs4886747 | UBE2Q2 | G | 2.6472 | 0.21784 | 5.6927E-34 |
| rs1047891 | CPS1 | A | -2.1358 | 0.17638 | 9.6162E-34 |
| rs9392859 | RREB1 | G | 2.7469 | 0.22831 | 2.465E-33 |
| rs10224210 | PRKAG2 | C | 2.1792 | 0.18228 | 6.177E-33 |
| rs10265221 | PRKAG2 | C | 2.1641 | 0.18112 | 6.6915E-33 |
| rs1477141 | HLF | G | 1.9524 | 0.16377 | 9.2578E-33 |
| rs4724799 | UNCX | G | 2.4406 | 0.20482 | 9.9089E-33 |
| rs2075252 | LRP2 | T | -2.2598 | 0.1908 | 2.3599E-32 |
| rs10254101 | PRKAG2 | T | 2.1253 | 0.1813 | 9.9197E-32 |
| rs4971066 | SLC50A1, EFNA1 | G | -2.6054 | 0.2226 | 1.2243E-31 |
| rs4575545 | MAFTRR | A | -2.0883 | 0.17846 | 1.2628E-31 |
| rs4698067 | CLNK | A | -2.1299 | 0.18319 | 3.0531E-31 |
| rs35732917 | MLXIPL | C | -2.1044 | 0.18135 | 3.9956E-31 |

* Linear closest gene is annotated by Plink v1.0 and 3DSNP v1.0 (https://www.omic.tech/3dsnp/) .

SNPs, single nucleotide polymorphisms; LASSO, Least Absolute Shrinkage and Selection Operator; SE, standard error.

**Table S7. Mean performance metrics of base classifiers in the train set across 5-fold cross-validation.**

| **Classifier** | **Model** | **AUC** | **Accuracy** | **Sensitivity** | **Specificity** |
| --- | --- | --- | --- | --- | --- |
| ADB | SNP | 0.634 (0.003) | 0.601 (0.023) | 0.588 (0.029) | 0.603 (0.031) |
|  | Clinical | 0.795 (0.001) | 0.696 (0.013) | 0.757 (0.018) | 0.687 (0.017) |
|  | **Clinical+SNP** | **0.821 (0.003)** | **0.711 (0.014)** | **0.789 (0.017)** | **0.699 (0.019)** |
| CAB | SNP | 0.602 (0.002) | 0.514 (0.026) | 0.654 (0.037) | 0.493 (0.036) |
|  | Clinical | 0.794 (0.001) | 0.688 (0.014) | 0.765 (0.023) | 0.677 (0.020) |
|  | **Clinical+SNP** | **0.803 (0.003)** | **0.696 (0.008)** | **0.770 (0.014)** | **0.685 (0.011)** |
| KNN | SNP | 0.538 (0.004) | 0.556 (0.124) | 0.491 (0.158) | 0.566 (0.165) |
|  | Clinical | 0.734 (0.001) | 0.716 (0.002) | 0.636 (0.004) | 0.728 (0.003) |
|  | **Clinical+SNP** | **0.572 (0.005)** | **0.645 (0.003)** | **0.430 (0.010)** | **0.676 (0.004)** |
| LGBM | SNP | 0.615 (0.004) | 0.556 (0.037) | 0.621 (0.046) | 0.547 (0.049) |
|  | Clinical | 0.789 (0.002) | 0.689 (0.010) | 0.758 (0.013) | 0.679 (0.013) |
|  | **Clinical+SNP** | **0.799 (0.001)** | **0.703 (0.016)** | **0.761 (0.022)** | **0.695 (0.022)** |
| Logistic Regression | SNP | 0.669 (0.003) | 0.615 (0.018) | 0.633 (0.021) | 0.612 (0.024) |
|  | Clinical | 0.793 (0.001) | 0.698 (0.009) | 0.750 (0.015) | 0.690 (0.013) |
|  | **Clinical+SNP** | **0.831 (0.002)** | **0.723 (0.007)** | **0.793 (0.011)** | **0.712 (0.009)** |
| Random Forest | SNP | 0.591 (0.002) | 0.532 (0.035) | 0.610 (0.044) | 0.520 (0.046) |
|  | Clinical | 0.788 (0.001) | 0.686 (0.019) | 0.758 (0.023) | 0.676 (0.025) |
|  | **Clinical+SNP** | **0.773 (0.002)** | **0.661 (0.017)** | **0.763 (0.020)** | **0.647 (0.022)** |
| XGB | SNP | 0.638 (0.003) | 0.575 (0.025) | 0.632 (0.038) | 0.567 (0.034) |
|  | Clinical | 0.803 (0.001) | 0.686 (0.01) | 0.782 (0.013) | 0.672 (0.014) |
|  | **Clinical+SNP** | **0.828 (0.002)** | **0.722 (0.011)** | **0.784 (0.013)** | **0.713 (0.015)** |

Values are mean (SD) across 5 folds. Performance metrics for models are seven single classifiers. Rows correspond to the model being tested. AUC, area under the receiver operating characteristic curve; LGBM, Light Gradient Boosting Machine; XGB, classical extreme gradient boosting; CAB, Categorical Boosting; ADB, Adaptive Boosting; KNN, K-Nearest Neighbour.

**Table S8. Performance metrics of stacked machine learning model.**

| **Dataset** | **Model** | **AUC (95% CI)** | **Accuracy (95% CI)** | **Sensitivity (95% CI)** | **Specificity (95% CI)** |
| --- | --- | --- | --- | --- | --- |
| Train set | SNP | 0.703 (0.700,0.705) | 0.652 (0.65,0.654) | 0.630 (0.625,0.635) | 0.655 (0.653,0.657) |
|  | Clinical | 0.822 (0.820,0.824) | 0.718 (0.716,0.72) | 0.777 (0.773,0.781) | 0.709 (0.707,0.711) |
|  | Clinical+SNP | **0.859 (0.857,0.861)** | **0.736 (0.735,0.737)** | **0.828 (0.825,0.832)** | **0.723 (0.721,0.724)** |
| Internal test set | SNP | 0.661 (0.656,0.666) | 0.579 (0.576,0.582) | 0.668 (0.659,0.677) | 0.566 (0.562,0.569) |
|  | Clinical | 0.802 (0.798,0.806) | 0.701 (0.698,0.704) | 0.761 (0.754,0.769) | 0.692 (0.689,0.695) |
|  | Clinical+SNP | **0.836 (0.832,0.839)** | **0.740 (0.737,0.743)** | **0.775 (0.768,0.783)** | **0.734 (0.731,0.737)** |
| External test set | SNP | 0.588 (0.576,0.600) | 0.567 (0.557,0.577) | 0.559 (0.542,0.576) | 0.572 (0.559,0.585) |
|  | Clinical | 0.756 (0.745,0.766) | 0.703 (0.693,0.712) | 0.682 (0.666,0.698) | 0.716 (0.704,0.728) |
|  | Clinical+SNP | **0.779 (0.769,0.788)** | **0.723 (0.714,0.732)** | **0.664 (0.648,0.680)** | **0.759 (0.748,0.770)** |

AUC, area under the receiver operating characteristic curve; CI, confidence interval.

**Table S9. Performance metrics of stacked machine learning model in the age subgroups of external test set.**

| **Subgroup** | **Model** | **AUC (95% CI)** | **Accuracy (95% CI)** | **Sensitivity (95% CI)** | **Specificity (95% CI)** |
| --- | --- | --- | --- | --- | --- |
| Age<40 | SNP | 0.586 (0.570,0.602) | 0.568 (0.555,0.581) | 0.557 (0.534,0.579) | 0.575 (0.559,0.591) |
|  | Clinical | 0.758 (0.745,0.772) | 0.701 (0.688,0.713) | 0.726 (0.707,0.746) | 0.688 (0.672,0.703) |
|  | Clinical+SNP | **0.789 (****0.776,0.801)** | **0.723 (0.711,0.735)** | **0.736 (0.717,0.756)** | **0.715 (0.701,0.730)** |
| Age≥40 | SNP | 0.589 (0.570,0.609) | 0.562 (0.559,0.565) | 0.613 (0.588,0.638) | 0.524 (0.502,0.546) |
|  | Clinical | 0.743 (0.726,0.759) | 0.680 (0.677,0.683) | 0.742 (0.719,0.765) | 0.634 (0.613,0.655) |
|  | Clinical+SNP | **0.764 (0.748,0.780)** | **0.705 (0.702,0.708)** | **0.736 (0.714,0.759)** | **0.682 (0.661,0.702)** |

AUC, area under the receiver operating characteristic curve; CI, confidence interval.

**Table S10. The Associations of ISHUA with Gout and other outcomes in the train set.**

| **Outcome** | **Model 1** | | **Model 2** | | **Model 3** | |
| --- | --- | --- | --- | --- | --- | --- |
|  | **HR (95% CI)** | ***P*** | **HR (95% CI)** | ***P*** | **HR (95% CI)** | ***P*** |
| Gout | 327.37 [271.60, 394.59] | <0.001 | 326.62 [269.74, 395.50] | <0.001 | 289.86 [239.85, 350.29] | <0.001 |
| AF | 12.38 [11.08, 13.84] | <0.001 | 11.87 [10.60, 13.28] | <0.001 | 11.51 [10.28, 12.88] | <0.001 |
| CAD | 13.41 [12.16, 14.79] | <0.001 | 12.05 [10.91, 13.30] | <0.001 | 12.21 [11.06, 13.48] | <0.001 |
| DmT2 | 52.80 [47.65, 58.51] | <0.001 | 45.61 [41.11, 50.60] | <0.001 | 48.43 [43.65, 53.72] | <0.001 |
| HF | 26.49 [23.04, 30.45] | <0.001 | 22.90 [19.88, 26.39] | <0.001 | 23.60 [20.49, 27.18] | <0.001 |
| Hypertension | 14.29 [13.13, 15.55] | <0.001 | 13.32 [12.23, 14.51] | <0.001 | 13.09 [12.02, 14.26] | <0.001 |
| ESRD | 340.22 [254.22, 455.29] | <0.001 | 287.17 [213.64, 386.01] | <0.001 | 310.22 [231.00, 416.62] | <0.001 |
| All-cause death | 7.56 [6.84, 8.35] | <0.001 | 6.19 [5.59, 6.85] | <0.001 | 6.41 [5.79, 7.09] | <0.001 |

Model 1 was unadjusted;

Model 2 was adjusted for smoking status, alcohol consumption, physical activity, and diet;

Model 3 was adjusted for lifestyle; Participants were categorized into three groups according to the number of healthy lifestyle factors: (1) unfavorable (0 or 1 healthy lifestyle factors), (2) intermediate (2 factors), and (3) favorable (3 or 4 factors).

CAD, coronary artery disease; HF, Heart failure; ESRD, end stage renal disease; DmT2, type 2 diabetes mellitus; OS, overall survival; HR, Hazard ratio; CI, confidence interval.

**Table S11. The Associations of ISHUA with Gout and other outcomes in the internal test set.**

| **Outcome** | **Model 1** | | **Model 2** | | **Model 3** | |
| --- | --- | --- | --- | --- | --- | --- |
|  | **HR (95% CI)** | ***P*** | **HR (95% CI)** | ***P*** | **HR (95% CI)** | ***P*** |
| Gout | 380.81 [259.76, 558.25] | <0.001 | 373.26 [252.78, 551.15] | <0.001 | 333.76 [226.60, 491.61] | <0.001 |
| AF | 13.42 [10.72, 16.80] | <0.001 | 12.77 [10.17, 16.03] | <0.001 | 12.49 [9.95, 15.67] | <0.001 |
| CAD | 13.94 [11.48, 16.93] | <0.001 | 12.90 [10.60, 15.71] | <0.001 | 13.04 [10.71, 15.86] | <0.001 |
| DmT2 | 52.65 [42.90, 64.62] | <0.001 | 44.70 [36.33, 54.99] | <0.001 | 47.02 [38.21, 57.85] | <0.001 |
| HF | 27.39 [20.73, 36.17] | <0.001 | 23.55 [17.76, 31.23] | <0.001 | 24.29 [18.32, 32.20] | <0.001 |
| Hypertension | 17.08 [14.42, 20.23] | <0.001 | 15.80 [13.32, 18.75] | <0.001 | 15.79 [13.31, 18.73] | <0.001 |
| ESRD | 420.32 [233.00, 758.26] | <0.001 | 360.06 [198.25, 653.96] | <0.001 | 397.63 [219.13, 721.54] | <0.001 |
| All-cause death | 8.12 [6.67, 9.88] | <0.001 | 6.83 [5.60, 8.34] | <0.001 | 6.98 [5.72, 8.51] | <0.001 |

Model 1 was unadjusted;

Model 2 was adjusted for smoking status, alcohol consumption, physical activity, and diet;

Model 3 was adjusted for lifestyle; Participants were categorized into three groups according to the number of healthy lifestyle factors: (1) unfavorable (0 or 1 healthy lifestyle factors), (2) intermediate (2 factors), and (3) favorable (3 or 4 factors).

CAD, coronary artery disease; HF, Heart failure; ESRD, end stage renal disease; DmT2, type 2 diabetes mellitus; OS, overall survival; HR, Hazard ratio; CI, confidence interval.

**Table S12. The Associations of High-Risk group with Gout and other outcomes in the train set.**

| **Outcome** | **Risk group** | **Events** | **Participants** | **Model 1** | | **Model 2** | | **Model 3** | |
| --- | --- | --- | --- | --- | --- | --- | --- | --- | --- |
|  |  |  |  | **HR (95% CI)** | ***P*** | **HR (95% CI)** | ***P*** | **HR (95% CI)** | ***P*** |
| Gout | Low-Risk | 539 | 149250 | Reference |  | Reference |  | Reference |  |
| Gout | High-Risk | 1927 | 69021 | 7.90 [7.18, 8.69] | <0.001 | 7.56 [6.87, 8.32] | <0.001 | 7.46 [6.78, 8.21] | <0.001 |
| AF | Low-Risk | 5939 | 148896 | Reference |  | Reference |  | Reference |  |
| AF | High-Risk | 5353 | 71629 | 1.94 [1.87, 2.01] | <0.001 | 1.91 [1.84, 1.98] | <0.001 | 1.90 [1.83, 1.97] | <0.001 |
| CAD | Low-Risk | 7641 | 145802 | Reference |  | Reference |  | Reference |  |
| CAD | High-Risk | 7080 | 67869 | 2.07 [2.00, 2.14] | <0.001 | 2.00 [1.94, 2.07] | <0.001 | 2.01 [1.94, 2.07] | <0.001 |
| DmT2 | Low-Risk | 4096 | 147810 | Reference |  | Reference |  | Reference |  |
| DmT2 | High-Risk | 6275 | 69832 | 3.39 [3.25, 3.52] | <0.001 | 3.25 [3.12, 3.38] | <0.001 | 3.28 [3.15, 3.41] | <0.001 |
| HF | Low-Risk | 2947 | 149754 | Reference |  | Reference |  | Reference |  |
| HF | High-Risk | 3267 | 72243 | 2.36 [2.24, 2.48] | <0.001 | 2.25 [2.14, 2.36] | <0.001 | 2.26 [2.15, 2.38] | <0.001 |
| Hypertension | Low-Risk | 15376 | 116095 | Reference |  | Reference |  | Reference |  |
| Hypertension | High-Risk | 10117 | 42748 | 1.92 [1.87, 1.97] | <0.001 | 1.88 [1.84, 1.93] | <0.001 | 1.87 [1.83, 1.92] | <0.001 |
| ESRD | Low-Risk | 328 | 150061 | Reference |  | Reference |  | Reference |  |
| ESRD | High-Risk | 676 | 72745 | 4.33 [3.79, 4.94] | <0.001 | 4.04 [3.54, 4.61] | <0.001 | 4.12 [3.60, 4.70] | <0.001 |
| All-cause death | Low-Risk | 8550 | 150143 | Reference |  | Reference |  | Reference |  |
| All-cause death | High-Risk | 6597 | 72963 | 1.62 [1.56, 1.67] | <0.001 | 1.52 [1.47, 1.57] | <0.001 | 1.54 [1.49, 1.59] | <0.001 |

Model 1 was unadjusted;

Model 2 was adjusted for smoking status, alcohol consumption, physical activity, and diet;

Model 3 was adjusted for lifestyle; Participants were categorized into three groups according to the number of healthy lifestyle factors: (1) unfavorable (0 or 1 healthy lifestyle factors), (2) intermediate (2 factors), and (3) favorable (3 or 4 factors);

Abbreviations: CAD, coronary artery disease; HF, Heart failure; ESRD, end stage renal disease; DmT2, type 2 diabetes mellitus; OS, overall survival; HR, Hazard ratio; CI, confidence interval;

**Table S13. The Associations of High-Risk group with Gout and other outcomes in the internal test set.**

| **Outcome** | **Risk group** | **Events** | **Participants** | **Model1** | | **Model2** | | **Model3** | |
| --- | --- | --- | --- | --- | --- | --- | --- | --- | --- |
|  |  |  |  | **HR (95% CI)** | ***P*** | **HR (95% CI)** | ***P*** | **HR (95% CI)** | ***P*** |
| Gout | Low-Risk | 135 | 37201 | Reference |  | Reference |  | Reference |  |
| Gout | High-Risk | 457 | 17235 | 7.46 [6.15, 9.03] | <0.001 | 7.15 [5.89, 8.67] | <0.001 | 7.06 [5.82, 8.56] | <0.001 |
| AF | Low-Risk | 1423 | 37135 | Reference |  | Reference |  | Reference |  |
| AF | High-Risk | 1318 | 17992 | 1.98 [1.84, 2.14] | <0.001 | 1.95 [1.81, 2.10] | <0.001 | 1.94 [1.80, 2.09] | <0.001 |
| CAD | Low-Risk | 1920 | 36378 | Reference |  | Reference |  | Reference |  |
| CAD | High-Risk | 1807 | 17025 | 2.09 [1.96, 2.23] | <0.001 | 2.04 [1.91, 2.18] | <0.001 | 2.04 [1.92, 2.18] | <0.001 |
| DmT2 | Low-Risk | 1022 | 36873 | Reference |  | Reference |  | Reference |  |
| DmT2 | High-Risk | 1580 | 17547 | 3.40 [3.14, 3.68] | <0.001 | 3.24 [3.00, 3.51] | <0.001 | 3.26 [3.01, 3.53] | <0.001 |
| HF | Low-Risk | 724 | 37342 | Reference |  | Reference |  | Reference |  |
| HF | High-Risk | 845 | 18092 | 2.47 [2.24, 2.73] | <0.001 | 2.36 [2.13, 2.61] | <0.001 | 2.37 [2.15, 2.62] | <0.001 |
| Hypertension | Low-Risk | 3703 | 28965 | Reference |  | Reference |  | Reference |  |
| Hypertension | High-Risk | 2535 | 10748 | 1.98 [1.89, 2.09] | <0.001 | 1.94 [1.85, 2.04] | <0.001 | 1.94 [1.84, 2.04] | <0.001 |
| ESRD | Low-Risk | 85 | 37413 | Reference |  | Reference |  | Reference |  |
| ESRD | High-Risk | 161 | 18249 | 3.95 [3.04, 5.14] | <0.001 | 3.73 [2.86, 4.86] | <0.001 | 3.82 [2.93, 4.97] | <0.001 |
| All-cause death | Low Risk | 2213 | 37438 | Reference |  | Reference |  | Reference |  |
| All-cause death | High Risk | 1666 | 18299 | 1.57 [1.47, 1.67] | <0.001 | 1.49 [1.40, 1.59] | <0.001 | 1.49 [1.40, 1.59] | <0.001 |

Model 1 was unadjusted;

Model 2 was adjusted for smoking status, alcohol consumption, physical activity, and diet;

Model 3 was adjusted for lifestyle; Participants were categorized into three groups according to the number of healthy lifestyle factors: (1) unfavorable (0 or 1 healthy lifestyle factors), (2) intermediate (2 factors), and (3) favorable (3 or 4 factors);

Abbreviations: CAD, coronary artery disease; HF, Heart failure; ESRD, end stage renal disease; DmT2, type 2 diabetes mellitus; OS, overall survival; HR, Hazard ratio; CI, confidence interval;
